# Capsule carbohydrate structure determines virulence in *Acinetobacter baumannii*

**DOI:** 10.1371/journal.ppat.1009291

**Published:** 2021-02-02

**Authors:** Yuli Talyansky, Travis B. Nielsen, Jun Yan, Ulrike Carlino-Macdonald, Gisela Di Venanzio, Somnath Chakravorty, Amber Ulhaq, Mario F. Feldman, Thomas A. Russo, Evgeny Vinogradov, Brian Luna, Meredith S. Wright, Mark D. Adams, Brad Spellberg

**Affiliations:** 1 Department of Molecular Microbiology & Immunology, University of Southern California, Los Angeles, California, United States of America; 2 Department of Medicine, Keck School of Medicine, University of Southern California, Los Angeles, California, United States of America; 3 Stritch School of Medicine, Loyola University Chicago, Maywood, Illinois, United States of America; 4 Division of Infectious Diseases, Department of Medicine, Jacobs School of Medicine and Biomedical Sciences, University at Buffalo, Veterans Administration, Buffalo, New York, United States of America; 5 Department of Molecular Microbiology, Washington University School of Medicine, St. Louis, Missouri, United States of America; 6 National Research Council Canada, Human Health Therapeutics Centre, Ottawa, Canada; 7 Rady Children’s Institute for Genomic Medicine, San Diego, California, United States of America; 8 The Jackson Laboratory for Genomic Medicine, Farmington, Connecticut, United States of America; 9 LAC+USC Medical Center, Los Angeles, California, United States of America; Emory University School of Medicine, UNITED STATES

## Abstract

*Acinetobacter baumannii* is a highly antibiotic-resistant bacterial pathogen for which novel therapeutic approaches are needed. Unfortunately, the drivers of virulence in *A*. *baumannii* remain uncertain. By comparing genomes among a panel of *A*. *baumannii* strains we identified a specific gene variation in the capsule locus that correlated with altered virulence. While less virulent strains possessed the intact gene *gtr6*, a hypervirulent clinical isolate contained a spontaneous transposon insertion in the same gene, resulting in the loss of a branchpoint in capsular carbohydrate structure. By constructing isogenic *gtr6* mutants, we confirmed that *gtr6-*disrupted strains were protected from phagocytosis *in vitro* and displayed higher bacterial burden and lethality *in vivo*. *Gtr6*+ strains were phagocytized more readily and caused lower bacterial burden and no clinical illness *in vivo*. We found that the CR3 receptor mediated phagocytosis of *gtr6+*, but not *gtr6*-, strains in a complement-dependent manner. Furthermore, hypovirulent *gtr6+* strains demonstrated increased virulence *in vivo* when CR3 function was abrogated. In summary, loss-of-function in a single capsule assembly gene dramatically altered virulence by inhibiting complement deposition and recognition by phagocytes across multiple *A*. *baumannii* strains. Thus, capsular structure can determine virulence among *A*. *baumannii* strains by altering bacterial interactions with host complement-mediated opsonophagocytosis.

## Author summary

*Acinetobacter baumannii* is one of the most antibiotic-resistant pathogens in clinical medicine and is responsible for a significant number of deaths worldwide. We found that a highly virulent strain contained a mobile piece of DNA in one of its capsule assembly genes which rendered the gene inactive and thus removed a single sugar from the bacterium’s complex outer carbohydrate capsule. When we inactivated the same gene in a non-virulent related strain, it became virulent, and when we repaired the non-functional gene the virulent strain became non-virulent. We then determined that this single sugar was critical for innate immune cells to recognize and phagocytose bacteria, and that the cells depended on the deposition of host complement proteins on the capsule to recognize the strains with this extra sugar. This finding provides new insight into *A*. *baumannii* pathogenesis and may inform the development of future therapies against this insidious pathogen.

## Introduction

For the past two decades, *Acinetobacter baumannii* clinical infections have been on the rise due to its facile antimicrobial resistance repertoire, catapulting the organism into the public health spotlight. Indeed, *A*. *baumannii* is now the top priority listed on the World Health Organization list of pathogens requiring new therapeutic strategies [1]. Causing approximately 45,000 infections in the US annually (1 million worldwide), it has an abnormally high mortality rate relative to other Gram-negative species [2]. Typically acquired nosocomially, *A*. *baumannii* resists desiccation, persists on surfaces, and is primarily seen in the critical care environment where many patients experience prolonged contact with invasive medical devices [3]. *A*. *baumannii* isolates exhibit resistance to multiple classes of antimicrobials, leaving certain strains treatable by few antimicrobial therapies and others altogether untreatable [4–6]. Together, these factors have made *A*. *baumannii* an intractable public health issue refractory to traditional infectious disease therapies and requiring further research into its interaction with the host immune system.

Previous work has uncovered the importance of innate immune effectors in responding to bloodstream and pulmonary infections, specifically of macrophages, neutrophils, and complement. An antibody raised against *A*. *baumannii* exopolysaccharide capsule mediated complete protection against a hypervirulent strain in murine models of bacteremia and aspiration pneumonia, with clearance occurring primarily through Fc-receptor mediated phagocytosis by macrophages and neutrophils [7]. In untreated mice, mortality primarily occurs via TLR-4 mediated toxicity and sepsis through the release of endogenous lipopolysaccharide (LPS), directly dependent upon bacterial density in the blood or lung [8]. A clear delineation of virulence has been established by strain type, with more than 99.9% of certain less-virulent strains being cleared by 3- to 4-log CFU/ml in blood in the first two hours, while more virulent strains persisted or even expanded in density in the presence of fully functional innate-immune system effectors. Triple depletion of macrophages, neutrophils, and complement induced the conversion of a hypovirulent, rapidly-cleared strain (ATCC 17978) into a hypervirulent strain capable of *in vivo* lethality similar to a hypervirulent clinical isolate (HUMC1) [7]. Thus, escape from innate immune effectors is a key driver of *A*. *baumannii* virulence.

Capsule is a potential driver of innate immune effector evasion. For example, genetic lesions in capsule assembly genes resulting in an acapsular phenotype typically result in absence of strain virulence *in vivo* [9,10]. Furthermore, sub-inhibitory concentrations of chloramphenicol increase capsule thickness in *A*. *baumannii*, and increase both virulence and resistance to innate immune killing [11]. Nevertheless, both virulent and avirulent strains can have a functioning capsule [2], suggesting that variations in capsule structures, rather than presence or absence of capsule alone, may drive strain virulence. Here we present a mechanistic link between capsule structure and *A*. *baumannii* virulence using a strain collection of clinical isolates with well-defined capsule loci.

## Results

### Capsule genetic locus and carbohydrate structure

**Fig 1:**
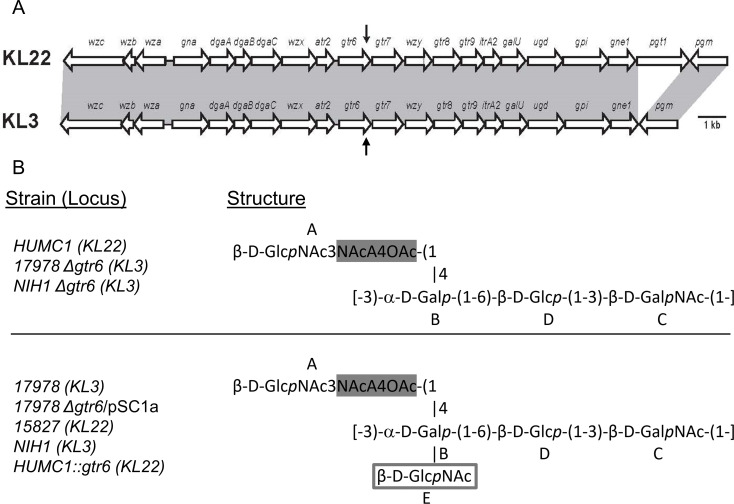
Capsular gene loci for *A*. *baumannii* KL3 and KL22 and capsular carbohydrate composition and linkage of KL22, and KL3 capsule locus strains. **(A)** Whole-genome sequencing of HUMC1 (a hypervirulent strain), 15827 (a hypovirulent strain) and ATCC 17978 (an avirulent strain) revealed distinct capsule loci organized into KL22 (HUMC1 and 15827) and KL3 (ATCC 17978) groups. KL22 differs from KL3 in that it contains an extra acetyltransferase gene *pgt1*, while HUMC1 (KL22) contains a transposon insertion sequence disruption in the coding region of the glycotransferase *gtr6* (downward black arrow). We then disrupted the *gtr6* gene in ATCC 17978 through the insertion of an antibiotic resistance cassette in its coding (upward black arrow), and then by replacing the entire gene with a defective copy from HUMC1. **(B)** (Top) Structural analysis of hypervirulent HUMC1 and the ATCC 17978 Δ*gtr6* mutant (KL3) revealed differential levels of acetylation at the A4 position marked in grey highlight (90% for *pgt1*^*+*^ HUMC1 and 50% for *pgt1*^*-*^ ATCC 17978 Δ*gtr6*). The two strains are isogenic at the capsule locus save for *pgt1*. (Bottom) Structural analysis of avirulent ATCC 17978 and hypovirulent 15827 (KL22) revealed the same *pgt1*-mediated difference in acetylation as well as an additional GlcNAc branch at position B (grey rectangle). Both KL22 and KL3 loci have a functioning *gtr6* gene.

We previously defined the *in vivo* virulence of several *A*. *baumannii* clinical isolates [7,8,12]. After sequencing these strains we identified several with defined and relatively conserved [13] capsule loci genetic elements and highly variable virulence [2] through analysis with the Basic Local Alignment Search Tool (BLAST) **(Table 1)**. *A*. *baumannii* HUMC1, a hypervirulent clinical blood and lung isolate, contains a KL22-type capsule locus type per the Kenyon classification [13]. ATCC 17978, a lab-adapted avirulent reference strain originally isolated from cerebrospinal fluid more than 50 years ago, is a KL3-type strain. Only two differences were found in the capsule loci of these strains, which exhibit vastly different *in vivo* virulence [14]. First was the presence of an extra gene (*pgt1*) near the end of the capsule locus in the KL22 type strain (HUMC1), and not in the KL3 strain (ATCC 17978). Second was a transposon insertion near the end of the *gtr6* coding region resulting in a truncated mRNA sequence in the hypervirulent strain, HUMC1 (**Fig 1A**). BLAST analysis of the *gtr6* insertion revealed it to be already classified as ISAba13, belonging to Insertion Family 5 and Group 903, and present in over 50 strains of *A*. *baumannii*, some of which were confirmed to be clinical isolates.

**Table 1:** Strains by Locus Classification, Genotype, and Phagocytosis Phenotype. All strains used in this study are described according to Kenyon classification capsule assembly locus type, genotype by *gtr6* and *pgt1*, and relative phagocytic potential.:: = chromosomal gene insertion, / = plasmid insertion, * = generated mutant.

Strain	Locus Type	*gtr6*	*pgt1*	Phagocytosis
**HUMC1**	KL22	**-**	**+**	Low
**NIH1**	KL22	**+**	**+**	High
**15827**	KL22	**+**	**+**	High
**ATCC 17978**	KL3	**+**	**-**	High
ATCC 17978 Δgtr6*	KL3	**-**	**-**	Low
ATCC 17978 Δ*gtr6*/pSC1a*	KL3	**+**	**-**	High
NIH1 Δgtr6*	KL22	**-**	**-**	Low
HUMC1::gtr6*	KL22	**+**	**+**	High

When these two differences between HUMC1 and ATCC 17978 capsule loci were evaluated in other KL22- and KL3-type strains, we found that strains with intact *gtr6* genes were readily phagocytosed [12] **(Table 1)**. In contrast, *pgt1* was present in strains that had both low uptake (HUMC1) and high uptake (15827 and NIH1), and could therefore not be principally responsible for phagocytic phenotype.

Translated BLAST analysis predicted the *gtr6* gene to most likely be a glycosyltransferase and *pgt1* to be a phosphoglycerol transferase or sulfatase. After extraction and purification of HUMC1, ATCC 17978, and 15827 capsular polysaccharides, proton nuclear magnetic resonance (^1^H-NMR) and two-dimensional NMR spectra were obtained for each strain to determine their structural configuration. All strains shared a core structure composed of a repeating subunit of α-d-galactose, β-d-glucose, and N-acetyl-β-d-galactosamine (Residues B, C, and D in **Fig 1B**). They also contained a single N-acetyl-β-d-glucosamine side chain branching off of Residue B that was differentially acetylated (Residue A), with 50% overall acetylation in *pgt1-* strains (ATCC 17978) versus 90% acetylation in *pgt1+* strains (HUMC1 and 15827). Strains with intact *gtr6* (ATCC 17978 and 15827) had an additional single sugar residue consisting of an N-acetyl-β-d-glucosamine (Residue E) branching off of Residue B. This residue was absent in the HUMC1 strain, which has a spontaneously disrupted *gtr6* gene, suggesting that the disruption or absence of *gtr6* led to loss of Residue E.

### Construction and comparison of isogenic strain pairs

To better understand the role of *gtr6* in virulence, we created a series of isogenic strain pairs and compared them for virulence *in vitro* and *in vivo*. Specifically, we disrupted *gtr6* in ATCC 17978 and NIH1; created a revertant strain of the *gtr6*-disrupted ATCC 17978 mutant by transforming it with a functioning *gtr6*-containing plasmid; and repaired the spontaneous transposon disruption of *gtr6* in HUMC1 with a functional copy from ATCC 17978. Capsule carbohydrate analysis of ATCC 17978 Δ*gtr6* revealed the loss of the N-acetyl-β-d-glucosamine residue seen in the wild type strain (residue E above) as well as the retention of 50% acetylation of residue A consistent with the absence of a *pgt1* gene in the mutant strain.

**Fig 2:**
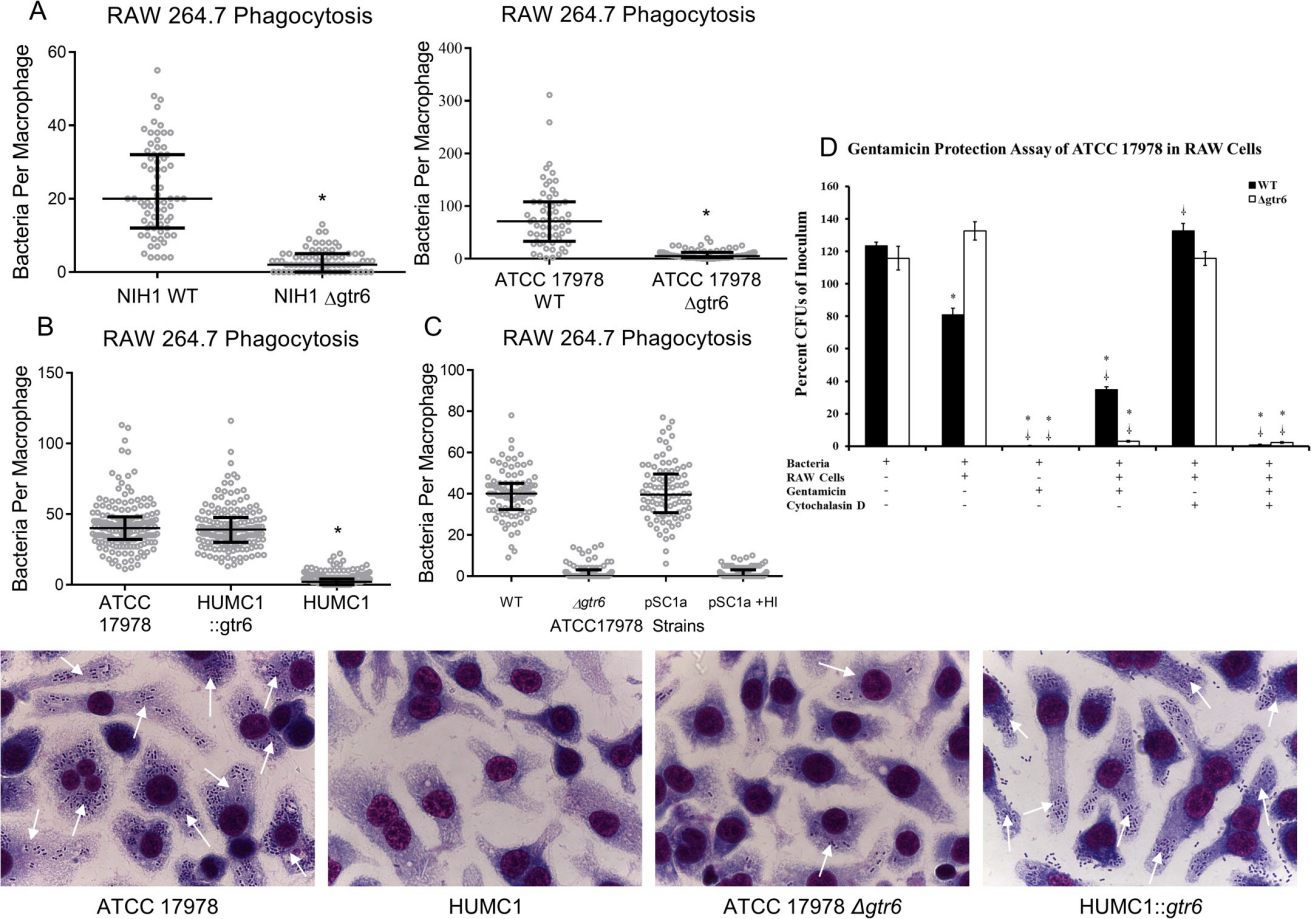
Macrophage phagocytosis of ATCC 17978 Δ*gtr6*, NIH1 Δ*gtr6*, and HUMC1::gtr6, gentamycin protection assay, and representative micrographs. **(2A)** RAW 264.7 cells were co-incubated with NIH1 (left) and ATCC 17978 (right) isogenic wild-type strains and Δ*gtr6* mutants. **(2B)** RAW 264.7 cells were co-incubated with ATCC 17978, the HUMC1::*gtr6* mutant strain with repaired *gtr6*, or wild-type HUMC1. **(2C)** RAW 264.7 cells were co-incubated with ATCC 17978 wild type, Δ*gtr6*, Δ*gtr6*/pSC1a (the knockout mutant with a plasmid-borne functional copy) in the presence of complement-active serum, and Δ*gtr6*/pSC1a in the presence of heat-inactivated serum. *p < 0.001. **(2D)** Gentamicin protection assay with RAW 264.7 cells and wild-type ATCC 17978 (black bars) or ATCC 17978 Δ*gtr6* (white bars). Cytochalasin D was added as an inhibitor of phagocytosis. Total bacteria plated for CFUs and expressed as a proportion of initial bacterial inoculum. * = significant vs. bacteria-only group, ⸸ = significant vs. bacteria + RAW 264.7 cell group. *,⸸ = p < 0.01 **(2E)** RAW 264.7 cells were incubated with ATCC 17978, HUMC1, ATCC 17978 Δ*gtr6*, and HUMC1::*gtr6*. Stained with Wright-Giemsa stain, total magnification is 1000x. Results are from two repeat experiments with duplicate samples in each. White arrows denote adherent or internalized bacteria.

As previously published, HUMC1 is intrinsically resistant to phagocytosis by neutrophils and macrophages, resulting in increased virulence in intravenous and intratracheal mouse infection models [14]. As for ATCC 17978 and NIH1, newly constructed strains with disrupted *gtr6* exhibited similar degrees of marked reduction in phagocytic uptake compared to their isogenic strains with intact *gtr6* (**Fig 2A**). In contrast, HUMC1 with repaired *gtr6* exhibited markedly increased uptake similar to all other strains with intact *gtr6* (**Fig 2B**). Representative micrograph images of RAW 264.7 bacterial uptake are reproduced in **Fig 2E**. Additionally, rescue of the ATCC 17978 *Δgtr6* mutant with a *gtr6*-containing plasmid restored phagocytic uptake (**Fig 2C)**. RNA sequencing analysis of wild-type HUMC1 and HUMC1::*gtr6* revealed no differential gene expression outside of the capsule locus (**S1A Fig**).

Bacterial internalization following adhesion was additionally confirmed through gentamicin protection assays using ATCC 17978 WT and ATCC 17978 *Δgtr6* (**Fig 2D)**. Specifically, gentamicin completely sterilized ATCC 17978 WT and *Δgtr6*, but was prevented from doing so when macrophages were co-cultured with the *gtr6+* strain but not the *Δgtr6* mutant, indicating macrophage uptake of the *gtr6+* strain (as gentamicin is active extracellularly but cannot reach bacteria inside macrophages). Furthermore, cytocholasin D, which abrogates phagocytosis, prevented macrophages from reducing *gtr6+* bacterial burden in culture and also prevented macrophages from protecting *gtr6+* bacteria from gentamicin-mediated sterilization.

**Fig 3:**
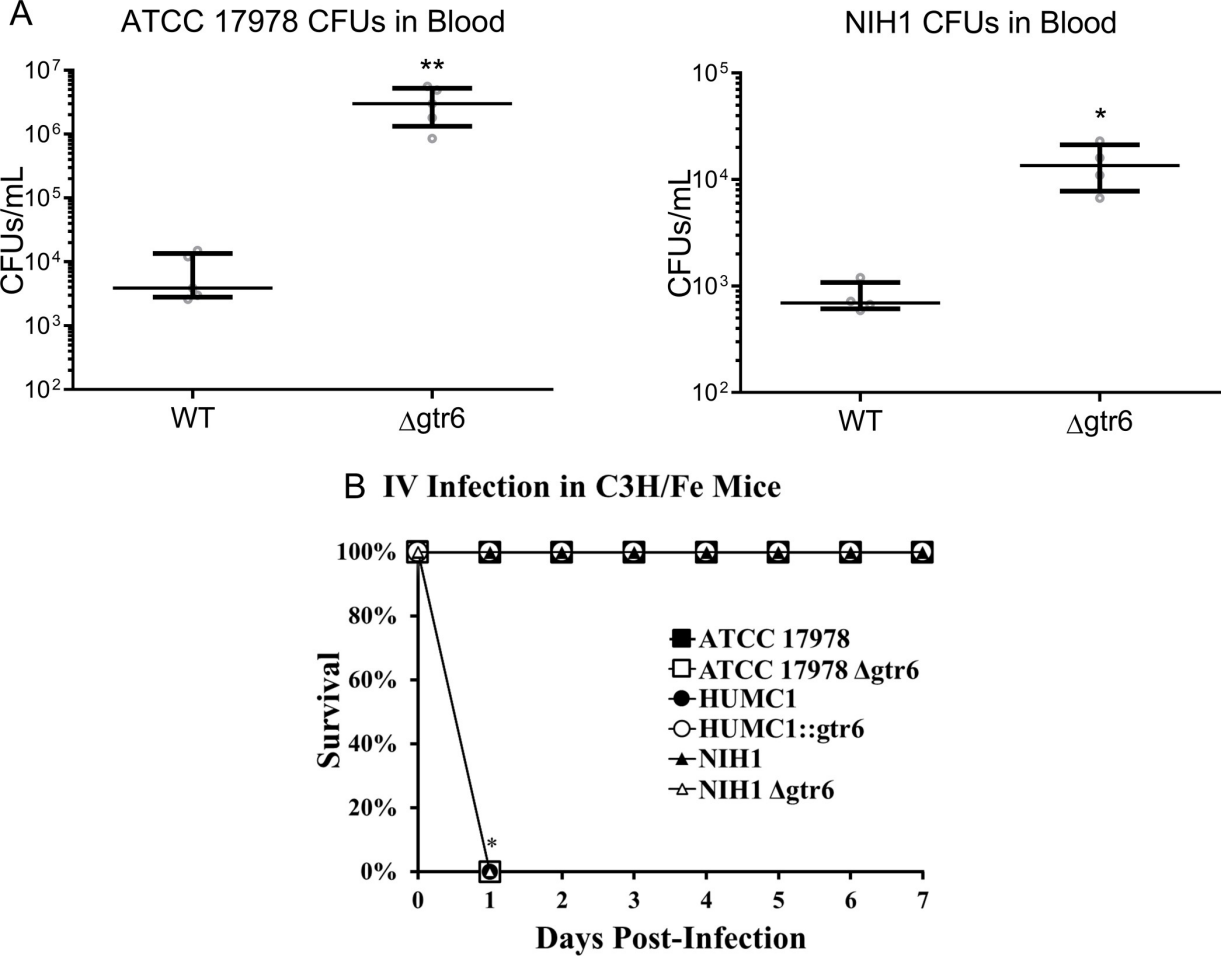
Bacterial blood burden and *in vivo* lethality by *gtr6* genotype. **(3A)** Bacterial burden in the blood at 1-hour post-infection with 1.0 ×10^8^ CFUs of ATCC 17978 WT and Δ*gtr6* (left) and NIH1 WT and Δ*gtr6* (right). *p < 0.001 **(3B)** C3HeB/Fe mice were infected intravenously with 2.4×10^8^ CFUs of ATCC 17978 (black squares), 8.3 ×10^7^ CFUs of ATCC 17978 Δ*gtr6* (white squares), 1.0 ×10^8^ CFUs of NIH1 (black circles) and NIH1 Δ*gtr6* (white circles), 2.9 ×10^7^ CFU of HUMC1 (black triangles), and 2.0 ×10^8^ CFUs of HUMC1::*gtr6* (white triangles). *p < 0.05, **p < 0.01. Wide bars denote median, error bars denote IQR. Experiments repeated once, n = 5 per group for *in vivo*.

When tested *in vivo* using a bacteremia mouse model, strains with disrupted *gtr6* resulted in markedly higher blood bacterial burden at 1-hour post-infection than those with intact *gtr6* (**Fig 3A**). We next compared the virulence of isogenic strain pairs *in vivo* and found that all strains with disrupted *gtr6* (ATCC 17978 Δ*gtr6*, NIH1 Δ*gtr6*, and HUMC1) were hypervirulent while all strains with intact *gtr6* (ATCC 17978, NIH1) were non-lethal (**Fig 3B**). Most notably, the *gtr6*-repaired mutant (HUMC1::*gtr6*) lost its virulence and was non-lethal at a 10-fold higher dose than the LD_100_ of wild type HUMC1 (**Fig 3B**).

### Mechanism of altered capsule structure on phagocytosis

Having established that *gtr6* disruption abrogates *A*. *baumannii* adhesion and subsequent phagocytosis *in vitro* and diminishes clearance and survivability *in vivo*, we next sought to determine how the capsule structure change mediated this effect.

**Fig 4:**
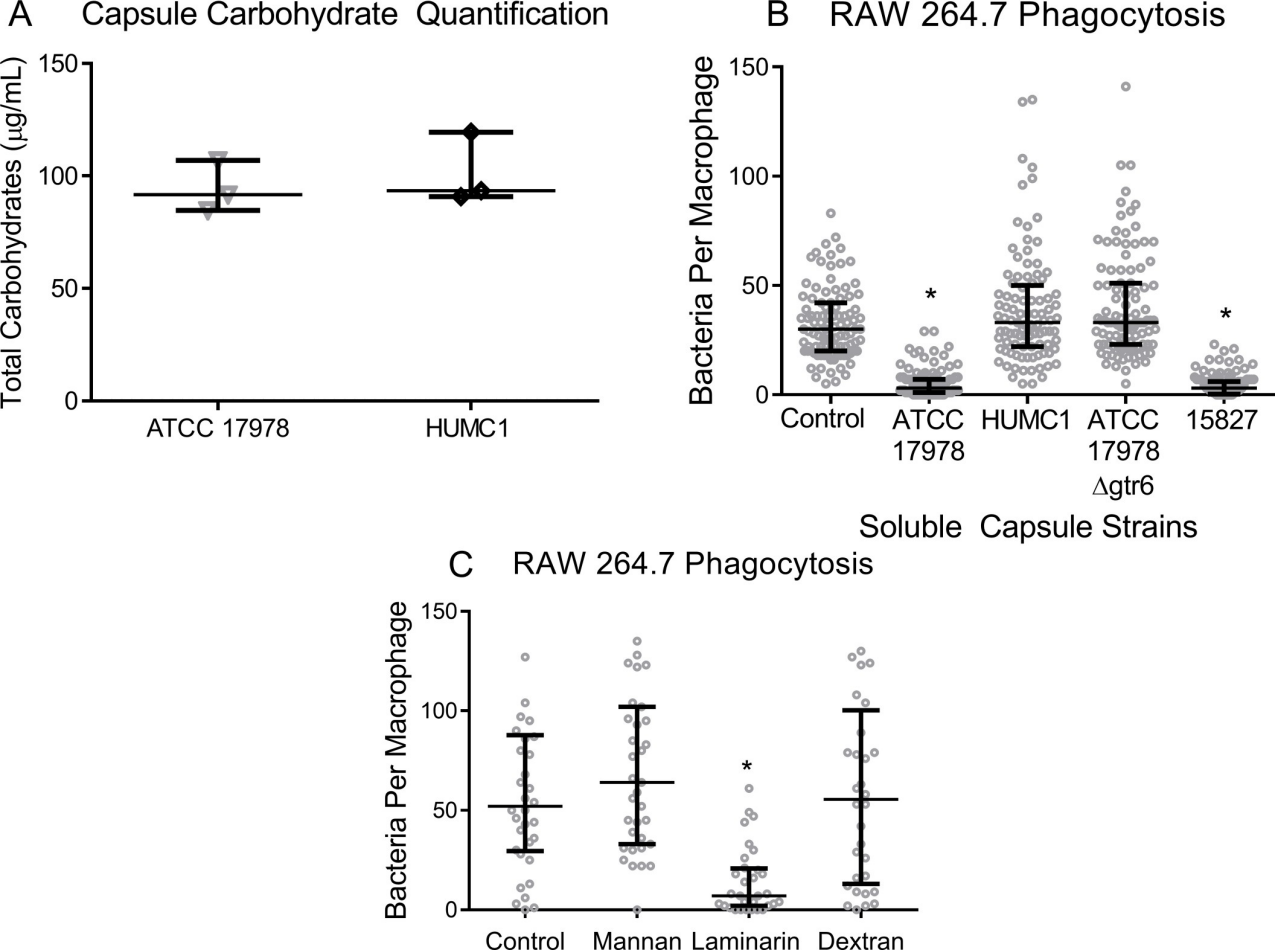
Quantification of capsule content, pre-incubation of phagocytes with purified bacterial capsule, and pre-incubation of phagocytes with soluble carbohydrates. (**4A**) 2.0×10^8^ CFU of ATCC 1778 and HUMC1 had total capsule carbohydrate capsule extracted in parallel and total carbohydrate content measured via phenol-sulfuric acid colorimetry. (**4B**) Incubation of macrophages and bacteria with purified capsule from *gtr6*^*+*^ (ATCC 17978, 15827) and *gtr6*^*-*^ (HUMC1, ATCC 17978 Δ*gtr6*) strains. Extract-free uptake was used as a control. *p < 0.0001 **(4C)** RAW 264.7 cells were pre-incubated with soluble mannan (0.5mg/mL), laminarin (0.5mg/mL), and dextran sulfate (0.1mg/mL) or an untreated control prior to co-incubation with ATCC 17978. *p < 0.0001. Two biological replicates for *in vitro*. Wide bars denote median, error bars denote IQR.

We first verified that *gtr6* did not affect capsule abundance by quantitatively measuring total carbohydrate content in capsule extracts **(Fig 4A)**. We subsequently sought to determine whether the *gtr6*-disrupted capsule actively inhibited phagocytosis or, conversely, *gtr6*-intact capsule promoted phagocytosis. We conducted mixed phagocytosis assays in which soluble capsule was extracted from strains and added to macrophage cultures in the presence of viable bacteria. We found that capsule extracted from *gtr6*-intact strains inhibited uptake of ATCC 17978 whereas capsule from *gtr6*-disrupted strains did not alter uptake of bacteria (**Fig 4B**). Thus, rather than actively inhibiting uptake, *gtr6*-disrupted strains produce a capsule structure that is unrecognizable by phagocytic receptors while not altering capsule abundance.

We next sought to identify which receptors were driving adhesion and phagocytosis of *gtr6*-intact strains. By pre-incubating macrophages with various carbohydrate targets of phagocytic receptors, we found that laminarin—but not mannan or dextran sulfate—inhibited the uptake of the normally highly phagocytosed strain ATCC 17978 (**Fig 4C**).

**Fig 5:**
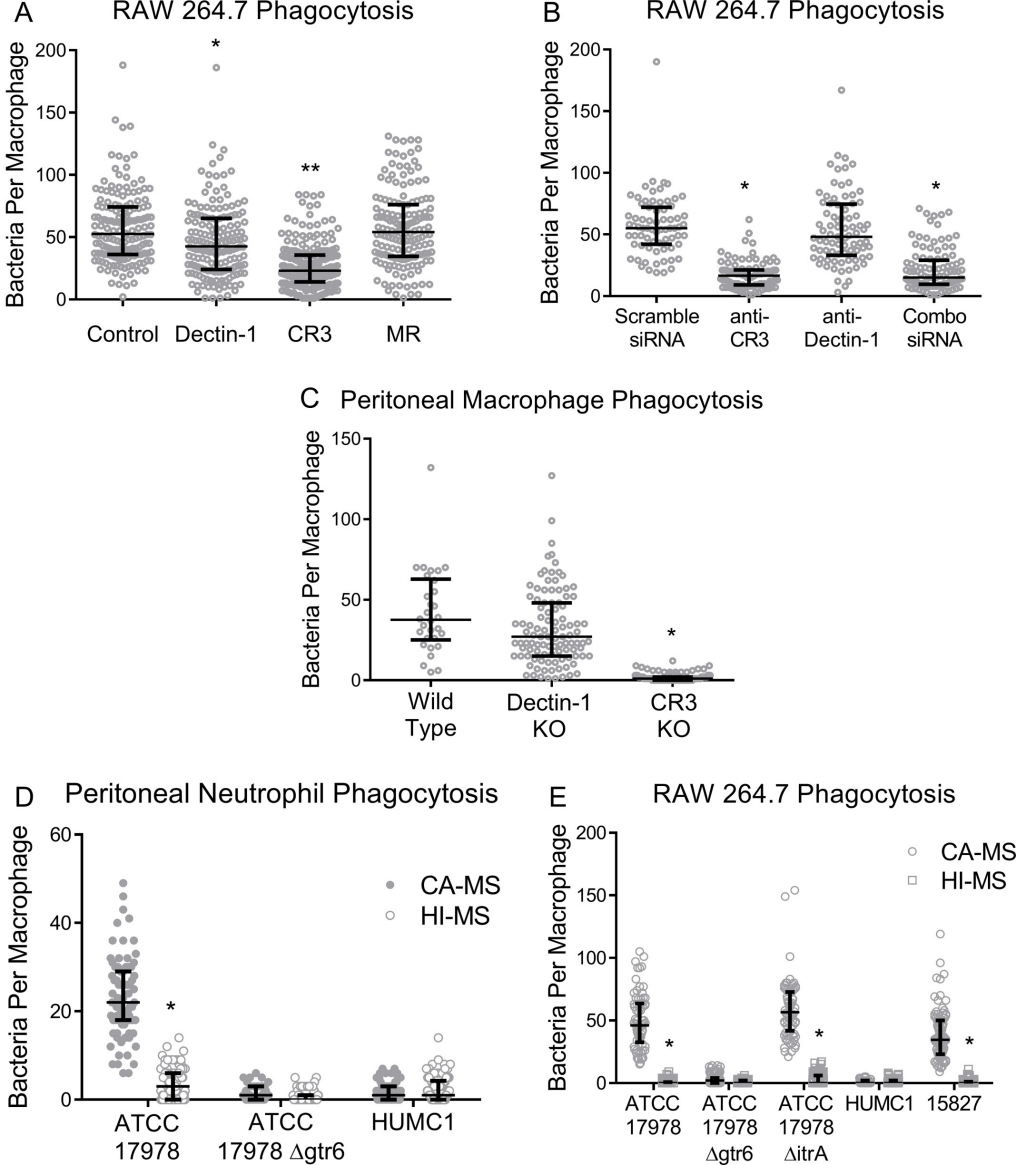
Receptor blockade with siRNA knockdown, antibody neutralization of beta-glucan receptors prior to bacterial uptake, and phagocytosis of bacteria by peritoneal macrophages and neutrophils. **(5A)** RAW 264.7 cells were pre-incubated with anti-Dectin-1, anti-CR3, anti-MR neutralizing monoclonal antibodies or an isotype control prior to co-incubation with ATCC 17978. *p < 0.0005, **p < 0.0001 **(5B)** Knockdown of Dectin-1 and/or CR3 in RAW 264.7 cells followed by incubation with ATCC 17978. *p < 0.0001 **(5C)** Primary peritoneally-elicited macrophages from C57BL/6 mice followed by phagocytosis assays with ATCC 17978. *p < 0.05, **p < 0.0001 **(5D)** Phagocytosis assays of ATCC 17978 with peritoneal neutrophils from wild-type mice with disruption of phagocytosis upon the addition of heat-inactivated serum (HI-S) or complement-active serum (CA-S). *p < 0.0001 **(5E)** Phagocytosis assays with RAW 264.7 macrophages with *gtr6*^*+*^ and capsule-free strains (ATCC 17978 WT, 15827, ATCC 17978 *ΔitrA*), and *gtr6*^*-*^ strains (ATCC 17978 *Δgtr6*, HUMC1), with complement active (CA-S) or heat-inactivated (HI-S) serum. *p < 0.0001. Experiments repeated once with two biological replicates. Wide bars denote median, error bars denote IQR.

Given that laminarin blocked phagocytosis of ATCC 17978 we next sought to block the known phagocytic receptors of laminarin using neutralizing monoclonal antibodies. Laminarin, a branched 1,3- and 1,6-linked β-glucan fungal sugar, is known to bind a number of mammalian C-type lectins including Dectin-1 and Complement Receptor 3 (CR3) [15]. We next performed phagocytosis assays with macrophages, *gtr6*-intact ATCC 17978, and neutralizing antibodies to identify which receptor interacted with capsular carbohydrate from *gtr6*-intact strains: anti-CR3 antibodies considerably decreased phagocytic uptake, anti-Dectin-1 antibodies modestly but statistically significantly decreased phagocytic uptake, and no decrease in phagocytosis was seen with anti-Mannose Receptor (MR) consistent with unaltered uptake upon pre-incubation with soluble mannan (**Fig 5A**).

To verify the involvement of CR3 in recognition of ATCC 17978, we knocked down CR3 and Dectin-1 mRNA in RAW 264.7 cells by siRNA transfection followed by phagocytosis assays. Consistent with prior siRNA results in this cell line [16], siRNA knockdown of CR3 resulted in a 50–75% receptor knockdown efficiency via ΔΔCt RT-qPCR (**S1B Fig).** Mimicking the effect of neutralizing antibodies, macrophages transfected with anti-CR3 siRNA showed a significant decrease in uptake of ATCC 17978, with a non-significant decrease in Dectin-1 and no additive effects with a dual Dectin-1/CR3 knockdown (**Fig 5B**).

Phagocytosis assays using peritoneal macrophages from Dectin-1- and CR3-knockout (KO) mice via 72-hour elicitation with Brewer thioglycolate medium yielded similar results. Specifically, CR3 null macrophages mediated drastically less uptake than macrophages from wild type or Dectin-1 KO animals (**Fig 5C)**. Shorter duration (24-hour) thioglycolate elicitation yielding peritoneal neutrophils showed genotypically similar results to macrophages (**Fig 5D**), and heat inactivation of complement by heating at 56°C for 30 minutes completely abrogated uptake in primary peritoneal neutrophils and RAW 264.7 macrophages (**Fig 5D and 5E**).

### The role of complement in mediating virulence

**Fig 6:**
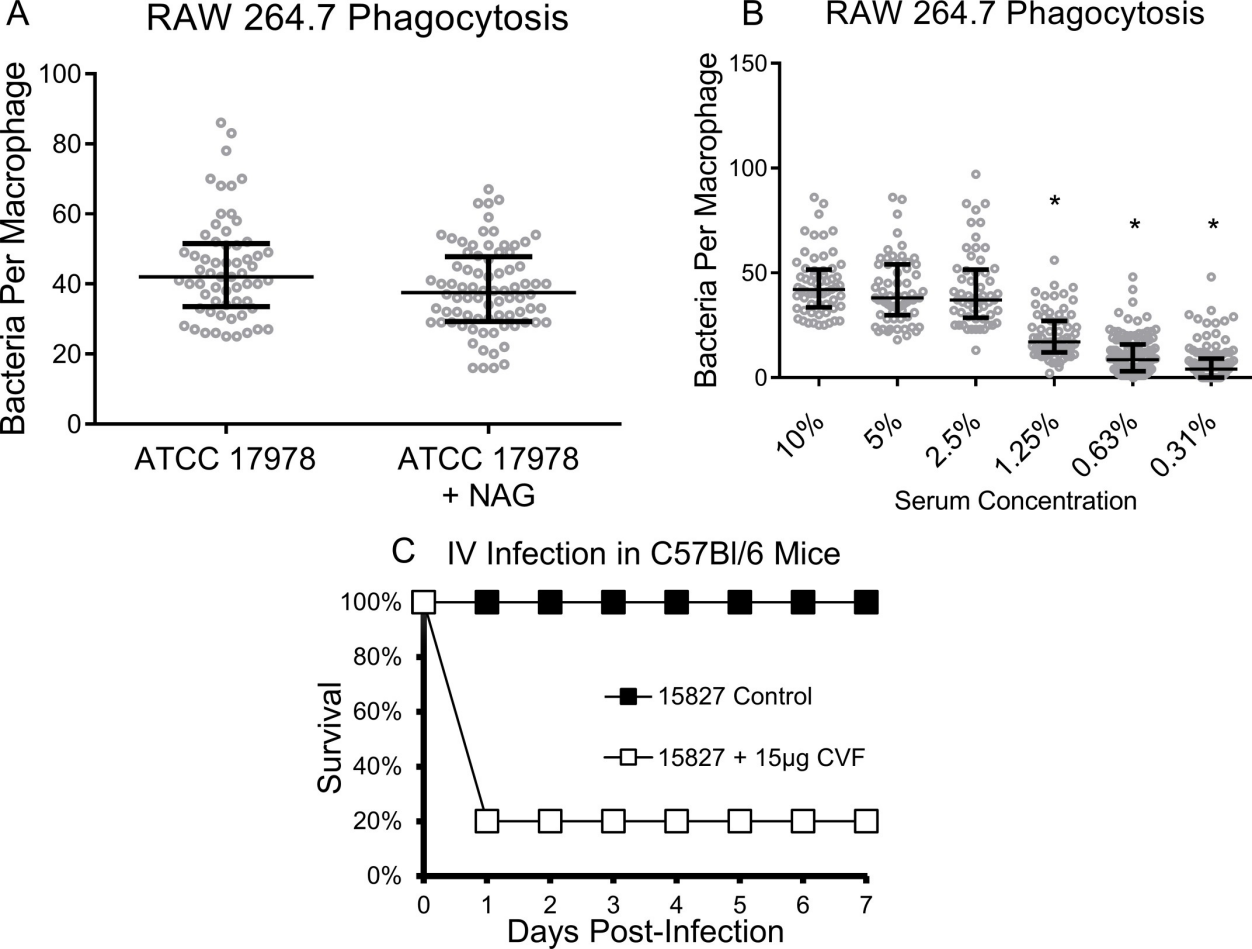
Phagocytosis in the presence of a lectin domain inhibitor, phagocytosis by macrophages in serially diluted serum, and infection of complement-depleted mice. **(6A)** Incubation of RAW 264.7 cells with ATCC 17978 in the presence of 100μg/mL GlcNAc (NAG), a CR3 lectin domain inhibitor. **(6B)** Serial two-fold dilutions of complement-active mouse serum in a RAW 264.7 cell phagocytosis assay with ATCC 17978. *p < 0.0001 **(6C)** Male C57BL/6 mice aged 10 weeks were infected intravenously with 2.0×10^8^ CFUs of 15827, with or without administration of 15μg cobra venom factor (CVF) 48 h prior to infection. *p < 0.001. Experiments repeated once, n = 5 per group for *in vivo* and two technical replicates for *in vitro*.

The dependence of phagocytes on CR3 and complement-active serum to uptake *gtr6*-intact strains suggested that complement deposition of iC3b is a primary driver of bacterial clearance. CR3 consists of both a complement-recognizing protein-binding domain and a carbohydrate-recognizing lectin domain [17], so we next sought to rule out any redundant effects between the two. To this end, we first antagonized the lectin-binding domain by pre-incubating macrophages with an inhibitory concentration [18] of soluble N-acetyl-d-glucosamine. Blockade of the CR3 lectin-binding domain in this manner did not alter uptake of bacteria in the presence of complement-active serum (**Fig 6A**). Serially diluting complement active serum demonstrated the dependence of macrophages on complement to uptake *gtr6*-intact ATCC 17978, with a significant loss of uptake occurring at ultra-low concentrations of complement of ≲1% (**Fig 6B**). Thus, even low amounts of complement were sufficient to drive CR3-mediated uptake of *A*. *baumannii*.

To establish the ability of complement to rescue mice from *A*. *baumannii* infection, we compared the concentrations of lethal inocula across strains in a murine bacteremia model, with mice depleted of complement using cobra venom factor (CVF) [19]. We previously found that *A*. *baumannii* strain 15827 was nonlethal at an inoculum of 2×10^8^ CFU whereas HUMC1—which has an identical KL22 capsule locus except for the *gtr6* disruption—was 100% lethal at an inoculum 10-fold lower [12]. 15827 also became highly lethal in mice depleted of complement relative to fully functional controls (**Fig 6C**).

**Fig 7:**
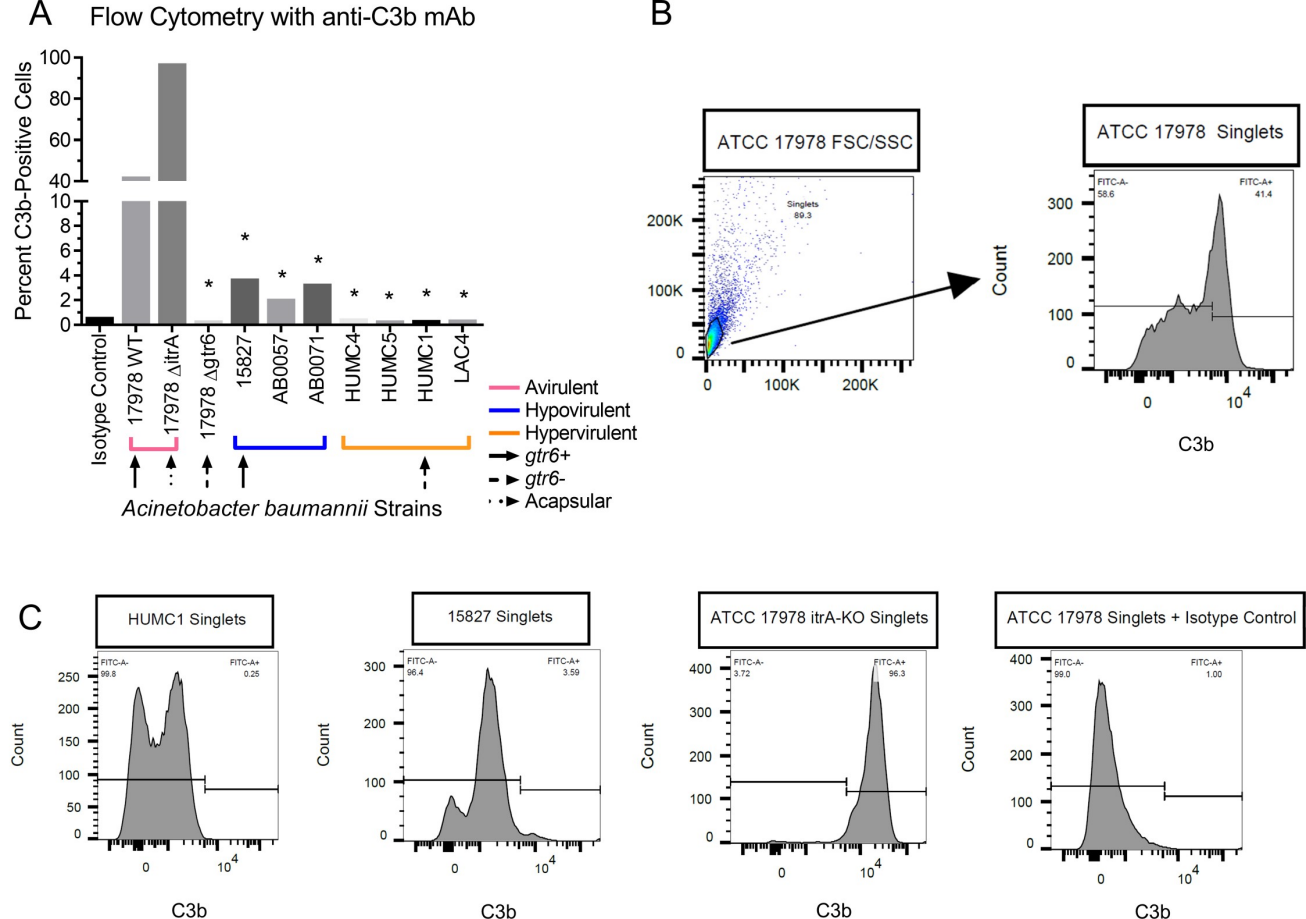
Flow cytometry of strains incubated with serum and anti-C3b antibodies. **(7A)** Flow cytometry of bacteria following incubation in 10% complement active serum followed by anti-C3b antibodies. Strains denoted by known virulence (brackets) as well as *gtr6* phenotype (upward arrows). *p* < 0.0001. **(7B)** Representative flow plot of initial forward and side scatter plot and sub-gating on single bacterial cells with FITC-A as the anti-C3b fluorophore. **(7C)** Representative histograms of anti-C3b fluorescent bacteria for HUMC1 (hypervirulent), 15827 (hypovirulent), ATCC 17978 Δ*itrA* (avirulent), and the isotype control. 20,000 events collected per condition for flow cytometry, gated for singlets via FSC/SSC, fluorescence gate set to exclude 99% of isotype control and copied across samples ran in parallel.

This led us to evaluate complement deposition on the bacterial surface. We first incubated bacterial strains with complement-active serum, followed by anti-C3b antibodies, and finally a fluorescent secondary antibody (**Fig 7A, 7B and 7C**). Flow cytometry revealed that C3b bound >40% of the *gtr6*+ ATCC 17978, >95% of an acapsular mutant (ATCC 17978 Δ*itrA*), and was almost undetectable on the *gtr6*− strain (ATCC 17978 Δ*gtr6*). Complement binding to other hypovirulent strains (15827, AB0057, AB0071) was considerably lower (2–5% events bound by C3b), but still 5- to 10-fold higher than the panel of hypervirulent strains (HUMC4, HUMC5, HUMC1, LAC4) which were nearly imperceptible (≤1% events bound by C3b). Thus, a small amount of complement deposition on the bacterial surface is sufficient to mediate phagocytic uptake *in vitro*. The role of C3 and C5 in phagocytosis were established via macrophage uptake assays of the strain panel in serum selectively depleted of C3 as well as C3/C5 in combination, as well as in entirely serum-free conditions. The presence of C3 was uniformly requisite for uptake (**S1C Fig**).

## Discussion

We have identified a single glycosyltransferase gene in the capsule locus that was capable of significantly modifying virulence in *A*. *baumannii*. A single β-d-Glc*p*NAc side chain alteration in the capsule dictated a hypovirulent versus hypervirulent phenotype in both wild-type and generated mutant strains. Strains lacking this key residue could not be readily phagocytized by innate immune effectors *in vitro*, nor be effectively cleared *in vivo*, and thus resulted in lethal infection. Conversely, strains possessing the *gtr6*-encoded capsular carbohydrate branch-point were readily adherent to immune cells, were phagocytosed, and were nonlethal *in vivo*.

Inserting both the transposon-disrupted HUMC1-derived *gtr6* gene as well as full disruption through replacement with an antibiotic cassette resulted in lack of phagocytosis and increased virulence, suggesting that the transposon insertion near the terminal coding region of *gtr6* in HUMC1 resulted in a complete functional knockout of the gene. Rescue of the ATCC 17978 *Δgtr6* mutant with a *gtr6-*containing plasmid reverted the phagocytosis phenotype, confirming that *gtr6* function, rather than polar effects of gene editing, were responsible for the phagocytosis phenotype seen in the generated mutants. RNA sequencing of the *gtr6*-disrupted wild type HUMC1 as well as the *gtr6+* HUMC1::*gtr6* rescue strain confirmed that the addition of *gtr6* did not change the expression levels of any genes outside of the capsule locus.

These results follow the molecular Koch’s postulates modified for loss-of-function driving virulence [20], indicating that the bacterial capsule is a primary driver of virulence, as demonstrated across multiple clinical isolates and isogenic strain pairs. BLAST analysis confirmed that this insertion element has previously been characterized as ISAba13 and is present in a variety of *A*. *baumannii* clinical isolates, and other work confirms that frequent transposon-mediated disruption of genes contributes significantly to *A*. *baumannii* virulence in the form of outer molecule structural variation [21], metabolic function, and antimicrobial resistance [22,23].

One limitation of the data is that we cannot definitively distinguish which step in phagocytosis is altered by the *gtr6* gene. However, it is likely that capsular alteration affects adhesion, which is the first step in the phagocytosis cascade. The ATCC 17978 *Δgtr6* mutant showed almost identical CFU levels in the bacteria-only and bacteria plus macrophage groups compared to a decrease in the bacteria plus macrophage group with wild type ATCC 17978, suggesting that the entire *Δgtr6* bacterial inoculum was present in the assay supernatant upon plating with no bacteria adherent to or sequestrated in the RAW 264.7 cells. Likewise, the addition of cytochalasin D to gentamicin-containing wells did not alter gentamicin’s effect on CFUs with the *Δgtr6* strain. This result suggests that gentamicin protection is mediated upstream of the cytochalasin target in the assay, which is actin-polymerization mediated phagocytosis, after adhesion had already occurred.

Multiple lines of evidence indicated CR3 as the primary receptor mediating uptake via complement deposited on the bacterial surface. As CR3 contains both a C-type lectin-binding domain that recognizes carbohydrates and a protein-binding domain that recognizes inactivated (but bound) complement factor 3b (iC3b) [24], both could have played a role in the recognition of *A*. *baumannii* [25]. However, heat-inactivating serum completely blocked bacterial uptake by both macrophages and neutrophils while incubation with a CR3 lectin domain inhibitor did not, indicating that *in vitro* phagocytosis by CR3 depended entirely on bound complement recognition by two innate immune effector cell types. Furthermore, serially diluting the serum present in macrophage uptake assays decreased bacterial uptake only at ≲1% serum. Thus, only a small amount of capsule-bound complement was necessary for recognition and phagocytosis. Selective depletion of C3 additionally prevented uptake as did entirely serum-free conditions. Furthermore, capsule structure drove complement deposition. Specifically, an acapsular Δ*itrA* mutant strain was virtually saturated via complement binding on its surface, hypovirulent strains with intact *gtr6* exhibited intermediate complement binding, and hypervirulent, *gtr6-*deficient strains (HUMC1 and ATCC 17978 *Δgtr6*) had very little C3b bound (≤1% events), commensurate with their resistance to phagocytosis and clearance from the blood.

Interestingly, the *gtr6*+/*pgt1*+ strain (15827) showed moderate levels of C3b binding (~5% events), less bound C3b than the *gtr6*+/*pgt1*− strain (ATCC 17978). These results suggested that *pgt1* may play a small role in protecting bacteria from complement opsonization, though insufficient for increasing strain virulence on its own. Previous work has described surface charge as being important in both promoting and inhibiting complement deposition on bacterial and artificial surfaces [26,27], and the higher acetylation in *pgt1+* strains may be minimally protective against complement deposition via this model by having an overall higher negative surface charge than *pgt1-* strains. The specific pathway by which *pgt1* differentially acetylates capsule remains unknown, as its predicted function as a phosphoglycerol transferase or sulfatase is not reflected in the KL22 structure. However, inconsistencies between its presence in the capsule locus and capsule structure have been described previously [28,29].

The *gna* gene, present in multiple strains including KL3 and KL22, is most likely responsible for the synthesis of Glc*p*NAcA from Glc*p*NAc while *dgaA*, *dgaB*, and *dgaC* (annotated as *mnnA-C* elsewhere) are responsible for the synthesis of Glc*p*NAc3NAcA [28], a modified side chain of which is present in both KL3 and KL22 but does not seem play a principal role in innate immune recognition given that both ATCC 17978 and HUMC1 contain this residue in their capsule structures (although its differential acetylation may play a minimal role as discussed above). Notably, the hypervirulent clinical isolate LAC-4, which showed almost no complement deposition in our binding assay, has had its capsule structure characterized as KL49 [29], is entirely free of branch points, and consists of repeating subunits of α-FucNAc, α- d-Glc*p*NAc, and 8eLeg5Ac7Ac [30]. While containing a number of insertion sequences that potentially contribute to virulence [31], the LAC-4 capsule locus is free of insertion elements unlike HUMC1.

In conclusion, virulence across multiple strains of *A*. *baumannii* is driven primarily by interactions between bacterial capsule and distinct host innate effectors. Specifically, complement plays an integral role in coordinating phagocytosis, with its degree of deposition varying based on capsular polysaccharide structure, as mediated by the functionality of a capsule assembly gene. Capsule changes that preclude complement deposition markedly decreased phagocytic uptake via the protein-binding domain of the CR3 receptor, preventing bacterial clearance and leading to host death. We did not identify other receptors on host cells that were functionally redundant with CR3. However, Dectin-1 may play a minor role in host uptake of bacteria consistent with previous studies examining the relative contributions of CR3 and Dectin-1 in the uptake of glucan-bearing particles [15,32,33].

Capsule is thus a major virulence factor for *A*. *baumannii*, but a variety of other factors have been implicated in virulence as well [2,34,35]. While the intravenous bloodstream infection model mimics the second most common clinical manifestation of *A*. *baumannii* (bacteremia) [2], it is not necessarily safe to extrapolate to other disease settings (e.g., pneumonia, wound infections, urinary tract infections), which may involve innate immune effectors that differ significantly from those present in the bloodstream. However, we have found that anti-capsular monoclonal antibody therapy is protective during pneumonia, suggesting capsule does play a major role in pathogenesis during lung infection [7].

In summary, these results indicate that anti-virulence strategies specifically targeting the *A*. *baumannii* capsule or promoting complement deposition on bacteria (for example by antibody-based therapy) are promising means to prevent or treat serious infections caused by this deadly pathogen. Future work should determine how prevalent disruptions in *gtr6*, or other capsular alterations, are in clinical isolates of *A*. *baumannii*, and whether or not *gtr6*-positive strains are capable of innate immune evasion through an alternative mechanism.

## Materials and methods

### Ethics statement

All animal work was conducted following approval (Protocol # 20750) by the Institutional Animal Care and Use Committee at the University of Southern California, in compliance with the recommendations in the Guide for the Care and Use of Laboratory Animals of the National Institutes of Health. Infected mice develop weight loss, ruffled fur, poor appetite, decreased ambulation, huddling behavior, and low body temperature. Mice were monitored at least twice daily for seven days. Mice that displayed huddling behavior and are poorly mobile were weighed once daily. Weight loss of greater than 15% pre-infection body weight triggered euthanasia via CO_2_ chamber and secondary cervical dislocation. Soft bedding and other enrichment devices were provided as recommended by the veterinary staff. Nutritional supplements such as hydrogel packs were provided as needed.

### Genome BLAST analysis

Genomes were retrieved from NCBI with the following GenBank accession numbers: LQRQ00000000.1 (HUMC1), JMNX00000000.1 (15827), CP000521.1 (ATCC 17978), GCA_000222225.2 (NIH1). Nucleotide BLAST comparison of their K capsule loci was performed by first aligning to *fkpA/lldP* and *ilvE/aspS* genes that flank the K locus [13], and differing genes were analyzed for structural homology to known proteins using translated BLAST at NCBI. The *gtr6* transposon insertion in HUMC1 (NCBI Reference Sequence NZ_LQRQ01000007.1, transposon gene ID: AWC45_RS01000) was entered intro PATRIC for BLAST analysis and identified as ISAba13, belonging to Insertion Family 5 and Group 903.

### Knockout mutant generation

ATCC 17978 Δ*gtr6* and NIH1 Δ*gtr6*, isogenic derivatives of ATCC 17978 and NIH1 respectively, were generated by allelic exchange as described previously [36,37] with the following strain-specific selection marker and electroporation condition modifications. Electrocompetent cells were grown to OD_600_ 0.4 in lysogeny broth (LB) containing 0.12 mM Bi(NO_3_)_3_ and 2.5 mM sodium salicylate at pH 7 to decrease capsule production[38] followed by three washes with ice-cold 10% glycerol; cells were resuspended in sterile water to 500-fold pre-wash concentration. Electroporation was performed at 1.8 kV, 200 Ω, and 25 μF in a 2-mm cuvette. As a first step, to facilitate allelic exchange, the recombinase-containing plasmid pAT02 was introduced into ATCC 17978 and NIH1 via electroporation and selection with 200 μg/mL and 500 μg/mL of carbenicillin respectively. For the subsequent generation of electrocompetent cells containing pAT02, 2 mM IPTG and the appropriate concentration of carbenicillin were added after an initial 45 min of growth. For the construction of ATCC 17978 Δ*gtr6* and NIH1 Δ*gtr6*, a PCR-generated fragment that contained a kanamycin resistance gene flanked by the first and last 126 bp of *gtr6* was amplified and gel-purified. This fragment (7.8 μg) was electroporated into ATCC 17978/pAT02 or NIH1/pAT02 and recombinants were selected on LB plates containing 40 μg/mL kanamycin. Successful gene disruption was confirmed by sequencing of PCR-generated amplicons using primers outside of the gene in question. A derivative cured of pAT02 was used for subsequent studies. Strains were maintained at -80°C in 50% glycerol-50% LB.

### HUMC1 mutant generation

#### Strains, plasmids and growth conditions

*Acinetobacter baumannii* strain HUMC1 was maintained in LB. Plasmids were maintained in *Escherichia coli* JM101 with requisite antibiotics at concentrations as follows, unless otherwise specified: hygromycin 100 μg/mL; chloramphenicol 20 μg/mL; carbenicillin 100 μg/mL; tetracycline 25 μg/mL. Plasmids used for the study are listed in **S1 Text**. *A*. *baumannii* HUMC1 being an XDR strain, was found to be resistant to ampicillin, however it was sensitive to tetracycline at high concentration (60 μg/mL).

#### Construction of pAT03a

*E*. *coli* JM101 was first transformed with pSIM5 encoding the λ-Red recombination system [37,39] and JM101/pSIM5 was further transformed with pAT03 (ampR). pAT03a possesses a gene for a site-specific recombinase (flippase) that was used downstream to excise the hygromycin antibiotic resistance gene cassette **(S2 Text)** from the recombinant clone of *A*. *baumannii* HUMC1::*gtr6*-hygromycin. The plasmid pAT03a (**S2A Fig**) was derived from pAT03, by exchanging the ampicillin resistance gene cassette with a tetracycline resistance gene cassette as follows. The tetracycline resistance gene cassette was amplified from the plasmid pBS-Tet^r^ (**S1 Text**) using Q5 High-Fidelity Master Mix (NEB) using primer sets TetF and TetR (**S3 Text**). The 200-μL PCR reaction contained 80 ng DNA pBS-Tet^r^ template and primers at 0.5 μM. The mix was divided equally into four tubes and the amplification was done as follows: initial denaturation at 98°C for 3 min followed by 35 cycles of 96°C for 10 s, 62°C for 30 s, 72°C for 75 s, and the final extension was done at 72°C for 5 min. Upon confirmation on a 1% agarose gel for the presence of the expected amplicon size (1.3 kb), the PCR product was digested with DpnI in order to remove cell-derived plasmid template from the PCR sample. The reaction mix (230 μL) contained 195 μL PCR product, 23 μL 2× reaction buffer and three units of FastDigest DpnI (Thermo), incubated at 37°C in a water bath for 1 h. The PCR product was then purified by Monarch PCR & DNA Cleanup Kit (NEB). Electrocompetent *E*.*coli* JM101/pSIM5/pAT03 were prepared by growing the strain at 30°C to OD_600_ 0.6–0.8 in 10 mL LB (chloramphenicol, carbenicillin). Once the OD_600_ was reached, the culture was transferred to a 42°C water bath for exactly 15 min to induce the λ-Red recombinase in pSIM5, followed by cooling on ice for 30 min. Subsequently, 9 mL culture was centrifuged at 8,000×*g* for 6 min in 1.7-mL centrifuge tubes at 4°C. The pellet was washed twice with 4 mL ice-cold 10% glycerol and pellets from two centrifuge tubes were combined in 400 μL ice-cold 10% glycerol. The pooled pellets were resuspended in 100 μL 10% glycerol and stored at -80°C. The electrocompetent cells were transformed with 500 ng linearized PCR product using a BioRad Pulse Controller at 2.5 kV, 25 μF, and 200 Ω. Following incubation at 30°C for 2.5 h, 100 μL culture was plated onto LB (tetracycline) and incubated at 30°C for up to 48 h. Tetracycline-resistant colonies were screened for successful exchange of the ampicillin resistance gene with the tetracycline resistance gene by PCR using primers CHCK5Tet and CHCK3Tet (**S3 Text**). The colonies were grown overnight in 2.5 mL LB (tetracycline) and 1 μL culture was added to the PCR mix (25 μL; 1× Taq Frogga mix (Frogga Bio), with primers at 0.2 μM) and the amplification was done as follows: initial denaturation at 98°C for 3 min followed by 25 cycles of 96°C for 10 s, 52°C for 30 s, 72°C for 1 min and the final extension was done at 72°C for 5 min. pSIM5 was cured from the strain by two cycles of growth at 42°C. pAT03a was isolated using the Monarch Plasmid Miniprep Kit (NEB) and sequenced to confirm the fidelity of the tetracycline resistance gene.

#### *Construction of A*. *baumannii HUMC1*::gtr6*-hygromycin*

First, electrocompetent HUMC1 was prepared as follows. Colonies from an overnight LB agar plate were mechanically harvested and resuspended in 1 mL of LB, 500 μL of which was inoculated into 250 mL LB broth and incubated at 37°C while shaking at 275 rpm. The culture was harvested at an OD_600_ of 0.40–0.45, distributed into two 250 mL bottles and pelleted at 8000 x g for 8 mins at 4°C. The pellets were resuspended in equal volumes of ice cold 10% glycerol, followed by another wash with 70 mL of 10% glycerol. Then the two pellets were combined, washed with 50 mL of 10% glycerol and resuspended in a final volume of 500 μL of 10% glycerol. The plasmid pAT04 (500 ng) (**S2B Fig**), which possesses the *A*. *baumannii* recombination (Rec_Ab_) system (**S1 Text**), was transformed into 100 μL of HUMC1 competent cells via electroporation (Biorad pulse controller at 1.8 kV, 25 μF, 200 Ω). Transformed clones, (selected on LB tetracycline), were confirmed for presence of pAT04 by colony-PCR using the primers CHCK5Tet and CHCK3Tet as described above and verified as HUMC1/pAT04.

Next, the hygromycin resistance gene cassette with FRT sites was synthesized by Integrated DNA Technologies. The cassette was delivered in a pUC57 background (**S2C Fig**). Since the *gtr6* neighborhood of *A*. *baumannii* strain 15827 has the identical sequence as that in HUMC1 and possess wild-type *gtr6*, purified Ab15827 DNA was used as template for amplification of *gtr6*, starting from the 5’ end of the ORF up to 100 bases flanking the 3’ end.

Then, a plasmid construct (**S2D Fig**) was designed and generated by Gibson cloning in which the *gtr6* gene was followed by the hygromycin-FRT cassette and housed in a pUC19 background. *gtr6*, hygromycin-FRT resistance cassette and the pUC19 plasmid were amplified by PCR separately (**S1 Text**). *gtr6* and the pUC19 were amplified using Q5 High-Fidelity Master Mix (NEB), as described previously. The PCR conditions for gtr6 were: initial denaturation at 98°C for 3 mins., followed by 35 cycles of 96°C for 10 secs, 60°C for 30 secs, 72°C for 2 mins and 15 secs and the final extension was done at 72°C for 5 mins and for pUC19 were: initial denaturation at 98°C for 3 mins followed by 35 cycles of 96°C for 10 secs, 58°C for 30 secs, 72°C for 2 mins and 15 secs and the final extension was done at 72°C for 5 mins. The hygromycin-FRT cassette was amplified using Phusion HotStart II DNA Polymerase (Thermo Scientific). The reaction mixture (200 μL) contained 80 ng of template (pSC2), 1X GC buffer, 0.5 μM of each primer, 0.2 mM of each dNTPs and 3% DMSO. The PCR conditions for hygromycin-FRT gene cassette were: initial denaturation at 98°C for 3 mins followed by 35 cycles of 96°C for 10 secs, 70°C for 30 secs, 72°C for 2 mins and the final extension was done at 72°C for 5 mins. The three linear PCR amplicons were then purified using Monarch PCR & DNA Cleanup Kit (NEB). Equimolar amounts of these purified linearized fragments were ligated and circularized using Gibson Assembly Cloning Kit (NEB) following manufacturer’s protocol with the resultant generation of pSC1. 10 μL of the Gibson mix was transformed into electrocompetent *E*. *coli* JM101as described previously. Recombinant clones (JM101/pSC1) were selected by resistance to hygromycin and ampicillin.

Next, the plasmid pSC1 (**S2D Fig**) was isolated from JM101/pSC1 to enable amplification of the chimeric *gtr6*-hygromycin-FRT cassette using the primers Gtr6-Hyg 5 and Gtr6-Hyg 3 (**S3 Text**) and Phusion HotStart II DNA Polymerase as described previously. The PCR conditions were: initial denaturation at 98°C for 3 mins followed by 35 cycles of 96°C for 10 secs, 68°C for 30 secs, 72°C for 2 mins and 15 secs and the final extension was done at 72°C for 5 mins. The amplified product was concentrated to 1 μg/μL. Five μg of the linear chimeric *gtr6*-hygromycin-FRT cassette was transformed into electrocompetent *A*. *baumannii* HUMC1/pAT04. Electrocompetent *A*. *baumannii* HUMC1/pAT04 was prepared as described above with the following modifications: after 45 mins of growth, 2mM IPTG (which induces the recombinase) was added to the culture used to generate electrocompetent cells; IPTG (2mM) was also added to 4 mL of LB during the revival of the transformed culture, post-electroporation. Recombinant clones were selected on LB hygromycin (500 μg/mL). The correct site of recombination for the chimeric *gtr6*-hygromycin-FRT cassette into the chromosome, was confirmed by PCR amplification of the flanking regions of the *gtr6* neighborhood (Gtr6-Hyg Internal 5 and Gtr6-Hyg Flanking 3). The loss of the transposes (as expected) was also confirmed by sequencing the recombinant *gtr6* gene. The pAT04 was cured by selecting the HUMCI::*gtr6*-hygromycin strain consecutively on LB hygromycin for three times. Loss of PCR amplification by CHK5Tet and CHK3Tet primers confirmed loss of pAT04.

#### Flippase mediated excision of hygromycin resistance cassette HUMC1::gtr6-hygromycin to create HUMC1::gtr6

Electrocompetent cells of pAT04-cured HUMC1::*gtr6*:-hygromycin were prepared as described above. The flippase encoding plasmid pAT03a (560ng) was transformed into HUMC1::*gtr6*-hygromycin via electroporation (100μL competent cells in a 0.2cm cuvette at 1.8 kV, 25 μF and 200 Ω). The cells were subsequently grown in 1mL of LB broth containing 2mM IPTG (to induce flippase expression) for 90 min. at 37°C, 275 rpm. Cell suspensions were plated on LB plates containing tetracycline at 20 μg/mL. Recombinant colonies of interest in which flippase-mediated excision of the hygromycin cassette occurred were identified as tetracycline resistant, hygromycin sensitive when screened on LB tetracycline and LB hygromycin (500 μg/μL) plates. Colonies with this phenotype were further screened for the absence of the hygromycin cassette via PCR (2xFrogga Mix, primers Gtr6-Hyg Internal 5 and Gtr6-Hyg Flanking 3 (**S3 Text**), 95°C– 2min, [95°C– 30sec, 53°C– 30sec, 72°C– 1:30 min] x25, 72°C– 10min, 4°C—hold). Several colonies identified as having lost the hygromycin cassette were grown consecutively 6 times without any selection pressure in order to cure pAT03a. Phenotypic sensitivity to tetracycline followed by subsequent physical confirmation of the loss of the tetracycline gene cassette via PCR (using primers CHK5Tet and CHK3Tet as described) confirmed the loss of pAT03a. One colony of HUMC1::*gtr6* was used for further study. Genomic DNA was extracted and 62 ng was used as template in a 25μL PCR reaction with outside primers 1128/1129 (**S3 Text**) (0.5nM each), dNTPs (0.2nM), 5% DMSO, GC buffer and Phusion Hotstart II DNA Polymerase (Thermo Scientific). The reaction was visualized on an agarose gel and the band of the expected size was gel purified using the Monarch Gel Extraction Kit (NEB). Sequence analysis confirmed that HUMC1::*gtr6* possessed the restored genotype.

#### *Construction of the ATCC 17978* Δgtr6*/pSC1a rescue plasmid*

In order to make pSC1, the *gtr6-hyg* chimeric cassette was inserted in the middle of the *lacZ* gene of puc19, where all of the gene except of 5’ end 32 bases, was deleted. However, the *gtr6* gene in pSC1 was devoid of its promoter and was not inducible. Additionally, as a small portion of the 5’ end of the *lacZ* gene remained, the *gtr6* gene could not be induced by the lac promoter either. Hence, we decided to delete the 5’ end fragment of *lacZ* from the *gtr6* upstream region and clone the 192 base pair long indigenous promoter region of *gtr6* upstream of the gene itself thus creating pSC1a. The plasmid pSC1 (**S2E Fig**) and the *gtr6* indigenous promoter sequence (192 bp) were PCR amplified (**S3 Text**). The linearized plasmid PCR product was purified with NEB PCR clean up kit using manufacturer’s protocol while the promoter region PCR product was gel purified by NEB Gel purification kit following manufacturer’s protocol. The linear fragments were subjected to Gibson cloning using NEB Gibson Cloning kit following manufacturer’s protocol and was transformed in to NEB 5α Competent *E*. *coli* cells. The recombinant clones were selected on LB Hygromycin (150μg/mL) agar plates. Putative clones were grown overnight in 5mL LB Hygromycin (150μg/mL) broth and 1uL was used to perform colony PCR with primers Gtr6-Hyg Internal 5&3 as described previously.

### RNA sequencing

RNA sequencing was performed via a commercial platform (Novogene Corporation Inc, Sacramento, CA). Bacterial cells were grown overnight in tryptic soy broth, sub-cultured to logarithmic phase in tryptic soy broth, and cell pellets snap-frozen in liquid nitrogen. Following RNA extraction, total RNA was quantified, checked for purity via spectrophotometer, checked for integrity, and quantified using the RNA 6000 assay on the Bioanalyzer 2100 system. 1 μg total RNA was used per sample and sequencing libraries were analyzed via an Illumina sequence platform. Following quality control, reads were mapped to the ATCC 17978 reference genome and differential gene expression was quantified using the DESeq2 R package.

### Phagocytosis assays

We utilized RAW 264.7 macrophage-like cells activated for 24 hours with interferon-γ (IFN-γ), a condition comparable to activation with LPS [40,41] and previously utilized to successfully phagocytose *A*. *baumannii* strains [7]. RAW 264.7 cells were passaged in Dulbecco’s Modified Eagle Medium (DMEM) (Gibco, Thermo Fisher Scientific, Waltham, MA USA #11875135) supplemented with 10% Fetal Bovine Serum (FBS) (Atlanta Biologicals Inc, Flowery Branch, GA USA #S11150) at 37°C with 5% CO_2_ to a minimum of three and no more than 15 passages. After washing and counting, a concentration of 5×10^5^ cells/mL were stimulated with 1 μg/mL IFN- γ (Peprotech, Rocky Hill, NJ USA #315-05-B) and deposited onto glass coverslips, followed by overnight incubation.

Where indicated, macrophages were incubated prior to the addition of bacteria for 30 min at 37°C and 5% CO_2_ with soluble carbohydrates or antibodies. To block uptake, 0.5 mg/mL Mannan (Sigma-Aldrich, St. Louis, MO USA #M7504-100MG), 0.5 mg/mL Laminarin (Sigma-Aldrich #L9634-500MG), 0.1 mg/mL Dextran Sulfate (Sigma-Aldrich #D4911-1G), or 10 mM EDTA (VWR, #82021–254) were added to cells prior to incubation with bacteria. To neutralize receptors, anti-Dectin-1 (Invivogen, San Diego, CA USA #mabg-mdect), anti-CR3 (Thermo-Fisher, #14-0181-82), and anti-MR (Invivogen, #Mab-hMR) antibodies were added at 1:200. Bacterial strains were grown in Tryptic Soy Broth (TSB) (VWR, Radnor, PA USA #90000–372) overnight at 37°C with shaking at 200 rpm, sub-cultured to logarithmic phase, washed three times in PBS, diluted to 2×10^8^ CFUs/mL based on OD_600_ measurements, and added to RAW 264.7 cells at a multiplicity of infection of 20:1 in Hanks’ Balanced Salt Solution (HBSS) (VWR, #45001–101) supplemented with 10% complement-active CD-1 mouse serum (Innovative Research Inc., Novi, MI USA). In the case of complement dilution, two-fold dilutions of complement-active mouse serum in PBS were generated and added to the assays, with the total assay volume remaining at 1 mL. When performing mixed capsule assays, 1 μL purified capsule from strains was added to the culture plate prior to adding bacteria. Culture plates were centrifuged at 300×*g* for 5 min and incubated for 1 h at 37°C with 5% CO_2_. Plates were washed three times in HBSS, stained with HEMA-3 stain (Thermo Fisher Scientific, #22–122911), and mounted on glass microscope slides with VectaMount AQ aqueous mounting solution (VWR, #H-5501). Macrophages were visualized at 1,000× total magnification under oil immersion on a Leica DMLS brightfield microscope (Leica Microsystems Inc., Buffalo Grove, IL USA). The total numbers of internalized bacteria in each fully visible phagocyte on the microscope field were manually counted.

### Gentamicin protection assays

RAW 264.7 cells were activated and prepared as described above, and co-incubated with ATCC 17978 bacteria at a 20:1 MOI, with and without 200 μg/mL gentamicin and/or 20 μg/mL cytochalasin D at 37°C. At the 1-hour timepoint supernatant in gentamicin-free wells was agitated by gentle pipetting to resuspend un-phagocytosed bacteria and 100uL taken for CFU plating. In gentamicin-containing wells, gentamicin was added at the 1-hour timepoint followed by incubation at 37°C for 30 minutes. The supernatant was removed, macrophages were washed twice with HBSS, and 0.5% sodium deoxycholate added to selectively lyse macrophages but not bacterial cells. Cells were scraped from the wells using a pipette tip and 100uL of supernatant were plated for CFU measurement of internalized bacteria.

### Bacterial capsule purification and quantification

Bacterial cells were grown in 10 mL TSB overnight, centrifuged at 4,000×*g* for 5 min, and resuspended in 200 μL TAE buffer. 400 μL Lysis Buffer (100 mM SDS, 50 mM Tris, 0.128 mM NaCl) was added and solutions mixed by inversion. 600 μL of 25:24:1 phenol:chloroform:isoamyl alcohol solution was added and the solution was vortexed vigorously for 2 min until cloudy white. Samples were heated at 65°C for 15 min on a dry heating block and centrifuged in a benchtop centrifuge at 10,000 rpm for 15 min at 4°C. The upper aqueous phase was transferred to a new 1-mL tube and 200 μL sterile water was added. 50 μL 3 M sodium acetate and 1 mL ice-cold ethanol were added and the solution was mixed slowly by inversion. The solution was then held at -80°C overnight. The capsule extract was then purified by adding 3 μL 10 mg/mL DNase and 3 μL 10 mg/mL RNase and incubated at 37°C for 45 min. 5 μL 20 μg/mL Proteinase K was then added and the solution was incubated at 56°C for 1 h. An equal volume of phenol-chloroform-isoamyl alcohol mix was added and the solution was vigorously vortexed for 30 s. The samples were centrifuged at 10,000 rpm for 15 min at 4°C and the aqueous phase was transferred to a new 1.7-mL tube. 193 μL 50 mM Tris, 7 μL 3 M sodium acetate, a 3-fold greater volume of ice-cold ethanol was added and the samples were placed at -80°C overnight. The samples were spun at 10,000 rpm in a benchtop centrifuge at 4°C for 30 min, and resuspended in 50 μL sterile water.

To quantify total capsule carbohydrate content, bacterial cells were prepared as above and diluted to OD_600_ 0.5 and plated to count CFUs. After extraction in parallel as described above, total carbohydrate content was assayed via colorimetry as described elsewhere [42,43] in 96-well plates in a plate reader set to detect absorbance at 315 nm.

### siRNA knockdown in RAW 264.7 cells

RAW 264.7 cells were passaged in RPMI Medium 1640 (Gibco, Thermo Fisher Scientific, Waltham, MA USA #11875135) supplemented with 10% FBS at 37°C with 5% CO_2_. 2.5×10^5^ cells were deposited onto glass coverslips in 6-well tissue-culture treated plates, centrifuged at 300×*g* for 5 min, and allowed to adhere via incubation at 37°C with 5% CO_2_ for 1 h. Lipofectamine RNAiMAX Reagent (Thermo Fisher Scientific, Waltham, MA US #13387) was diluted in Opti-MEM Reduced Serum Medium (Thermo Fisher Scientific #31985062), and mixed 1:1 with anti-Dectin-1, anti-CR3, or scramble Mouse Silencer Select siRNA (Thermo Fisher Scientific #430817) diluted in Opti-MEM Reduced Serum Medium per manufacturer recommendations. siRNA-lipid complexes were added to wells with RAW 264.7 cells at 12.5 pmol and incubated for 24 h at 37°C with 5% CO_2_. Cells were then activated with 1 μg/mL IFN-γ, incubated for a further 24 h, and macrophage uptake assays were performed as above. To verify siRNA knockdown efficiency, CR3 or scramble siRNA knockdown was performed as described above, total RNA extracted, converted to cDNA, and finally measured via ΔΔCt RT-qPCR and expressed as a percentage of knockdown efficiency compared to the housekeeping gene GAPDH.

### Harvesting of elicited peritoneal phagocytes

3.8% Brewer Thioglycollate Broth was prepared by suspending 38 g Brewer Thioglycollate Medium (Sigma-Aldrich #B2551) in 1 L distilled water and sterilized by autoclaving at 121°C for 15 min. Male wild-type C57BL/6 mice, Dectin-1 KO, and CR3-KO mice (The Jackson Laboratory, Bar Harbor, ME) were injected intraperitoneally with 2 mL thioglycolate broth and peritoneal fluid was harvested: 72 h post-injection for macrophages; 24 h post-injection for neutrophils [44,45]. For harvesting, 5 mL warm PBS was injected directly into the peritoneum after euthanasia and aspirated. Suspended cells were then washed and resuspended in DMEM supplemented with 10% FBS. Cells were then incubated in T75 tissue-culture flasks for 2 h at 37°C with 5% CO_2_ to allow for adhesion. Non-adherent cells were removed by washing twice with warm DPBS, and adherent cells were resuspended in DMEM with 10% FBS followed by phagocytosis assays as described above.

### *In Vivo* infection model

Bacterial cultures were grown to logarithmic phase and washed as described previously[7]. Cultures were diluted so that 250 μL contained the target inoculum, which varied by strain and experiment. For lethal concentration and CFU experiments, male C3HeB/Fe mice aged 8–12 weeks were purchased from The Jackson Laboratory. For all knockout mouse experiments, male mice aged 8–12 weeks on a C57BL/6 background (strain # 003991 for CR3 KO and # 012337 for Dectin-1) along with wild-type controls were purchased from The Jackson Laboratory. Mice were briefly warmed under a heat lamp to dilate tail veins and 250 μL bacterial inocula were injected into the lateral tail vein. Mice were either monitored for survival with a moribundity endpoint in accordance with IACUC protocol or were euthanized following the administration of ketamine/xylazine and heparin per manufacturer instructions. Blood was collected from euthanized animals via cardiac puncture and serial dilutions plated on TSA for enumeration of CFUs. For cobra venom factor (CVF), 15 μg recombinant CVF resuspended in 200 μL PBS was injected intraperitoneally 48 hours prior to infection.

### Bacterial flow cytometry

Bacterial cultures were grown to logarithmic phase and washed as described previously. 1×10^7^ CFU were incubated with 10% complement-active mouse serum for 1 h at 37°C, washed three times with PBS, incubated with antibodies against mouse complement factor C3b (Thermo Fisher, clone 6C9) or an isotype control for 30 min, washed three times with PBS, and incubated with a secondary fluorescent antibody followed by three washes. Samples were then resuspended in FACS buffer and run on a Becton-Dickinson FACS Canto II flow cytometer, collecting 20,000 events per sample and gating on single cells with positive gates established at a fluorescence excluding 99% of the isotype control samples.

### Statistics

All *in vitro* experiments were performed with one biological replicate and were repeated once. For phagocytosis assays, five images were taken per coverslip and all cells within each image were counted. Median bacteria per macrophage were measured and non-parametric Mann-Whitney statistical tests were performed. For flow cytometry, all experiments were repeated once and 20,000 events per sample were collected. Fluorescence gates were established by excluding 99% of isotype control events. Statistical significance of proportions by positive and negative fluorescence was established via Chi-square contingency tests. *In vivo* experiments consisted of n = 5 animals per condition and were repeated once. Replicates were pooled and statistical significance was established via log-rank (Mantel-Cox) survival tests. All statistical tests were generated using Prism GraphPad 6 software.

## Supporting information

S1 Fig RNA Sequencing of HUMC1, SiRNA knockdown efficiency of CR3 in RAW 264.7 cells and phagocytosis assays with complement-depleted serum. **(A)** RNA sequencing of wild-type HUMC1 and HUMC1::*gtr6* showed no differential gene expression. **(B)** RAW 264.7 cells were incubated with anti-CR3 or scramble siRNA and knockdown efficiency measured via ΔΔCt RT-qPCR vs. the GAPDH housekeeping gene. **(C)** RAW 264.7 cells were incubated with ATCC 17978 in normal serum, in serum-free conditions, in serum selectively depleted of C3, and serum pre-treated with 15μg/mL cobra venom factor to deplete C3 + C5. * = p < 0.01.
(TIF)

S2 Fig Plasmids synthesized for mutant generation. For the generation of the HUMC1::*gtr6* mutant, plasmids **(A)** pAT03a-Tet, **(B)** pAT04, **(C)** pSC2, **(D)** pSC1 and **(E)** pSC1a were all synthesized as described in the Materials and Methods section.
(TIF)

S1 Text List of all plasmids used in mutant generation. Plasmid name, drug marker, function, and origin are listed.
(DOCX)

S2 Text Sequence of hygromycin resistance cassette for mutant generation. The cassette includes the FRT site (red), promoter site for hygromycin (green), and the hygromycin resistance gene (blue).
(DOCX)

S3 Text List of all primers used for mutant generation. Primer name, description, and sequence are listed. Underline–first/last 126bp of the *gtr6* ORF at the 5’ end.
(DOCX)
