# Supplementary figures and images for: Capsule carbohydrate structure determines virulence in Acinetobacter baumannii
Source: PLoS Pathog. 2021 Feb 2;17(2):e1009291. doi: 10.1371/journal.ppat.1009291 (PMC7880449; doi:10.1371/journal.ppat.1009291)

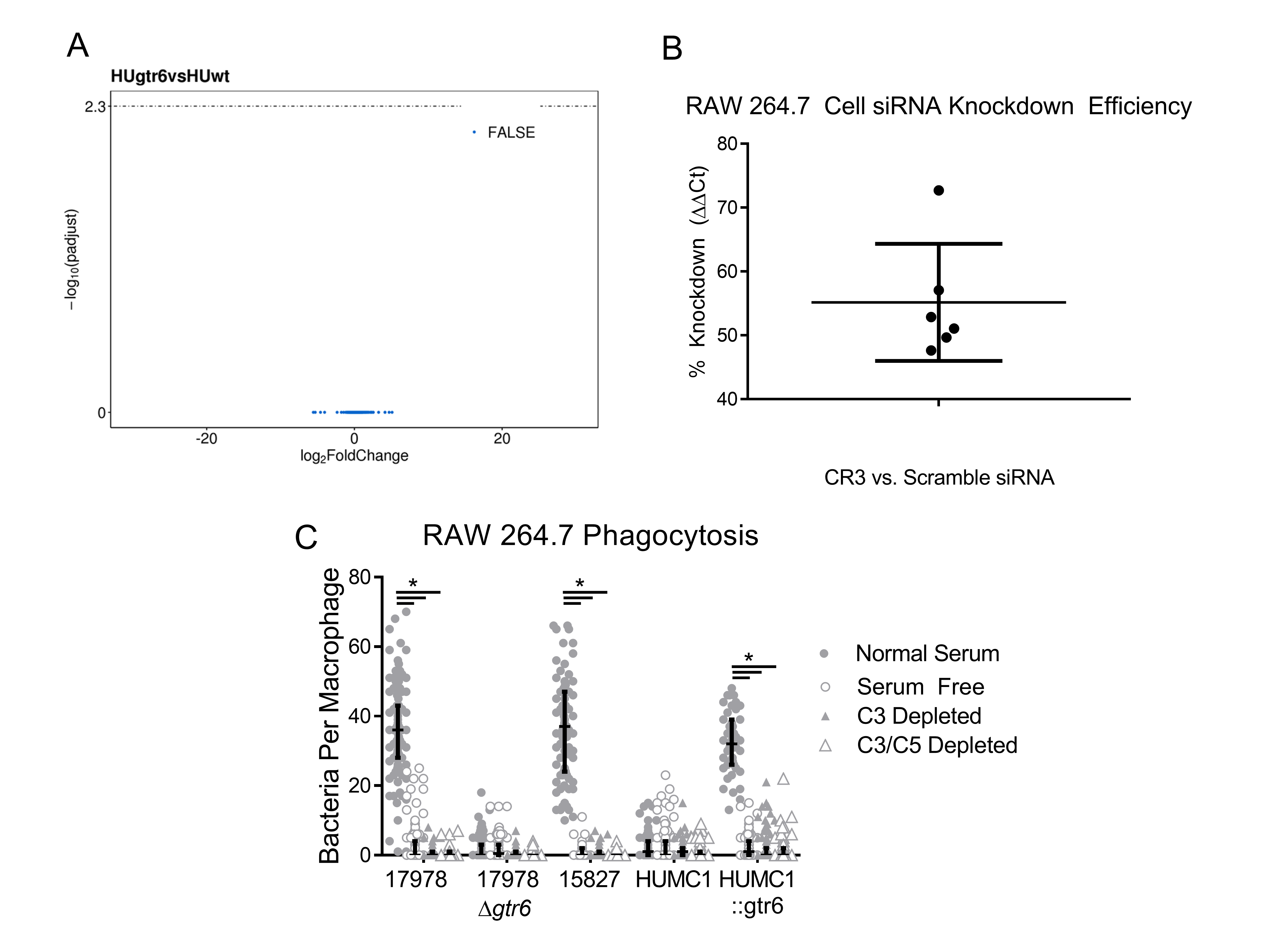

Supplement: S1 Fig — (A) RNA sequencing of wild-type HUMC1 and HUMC1::gtr6 showed no differential gene expression. (B) RAW 264.7 cells were incubated with anti-CR3 or scramble siRNA and knockdown efficiency measured via ΔΔCt RT-qPCR vs. the GAPDH housekeeping gene. (C) RAW 264.7 cells were incubated with ATCC 17978 in normal serum, in serum-free conditions, in serum selectively depleted of C3, and serum pre-treated with 15μg/mL cobra venom factor to deplete C3 + C5. * = p < 0.01. (TIF) [file ppat.1009291.s001.tif]

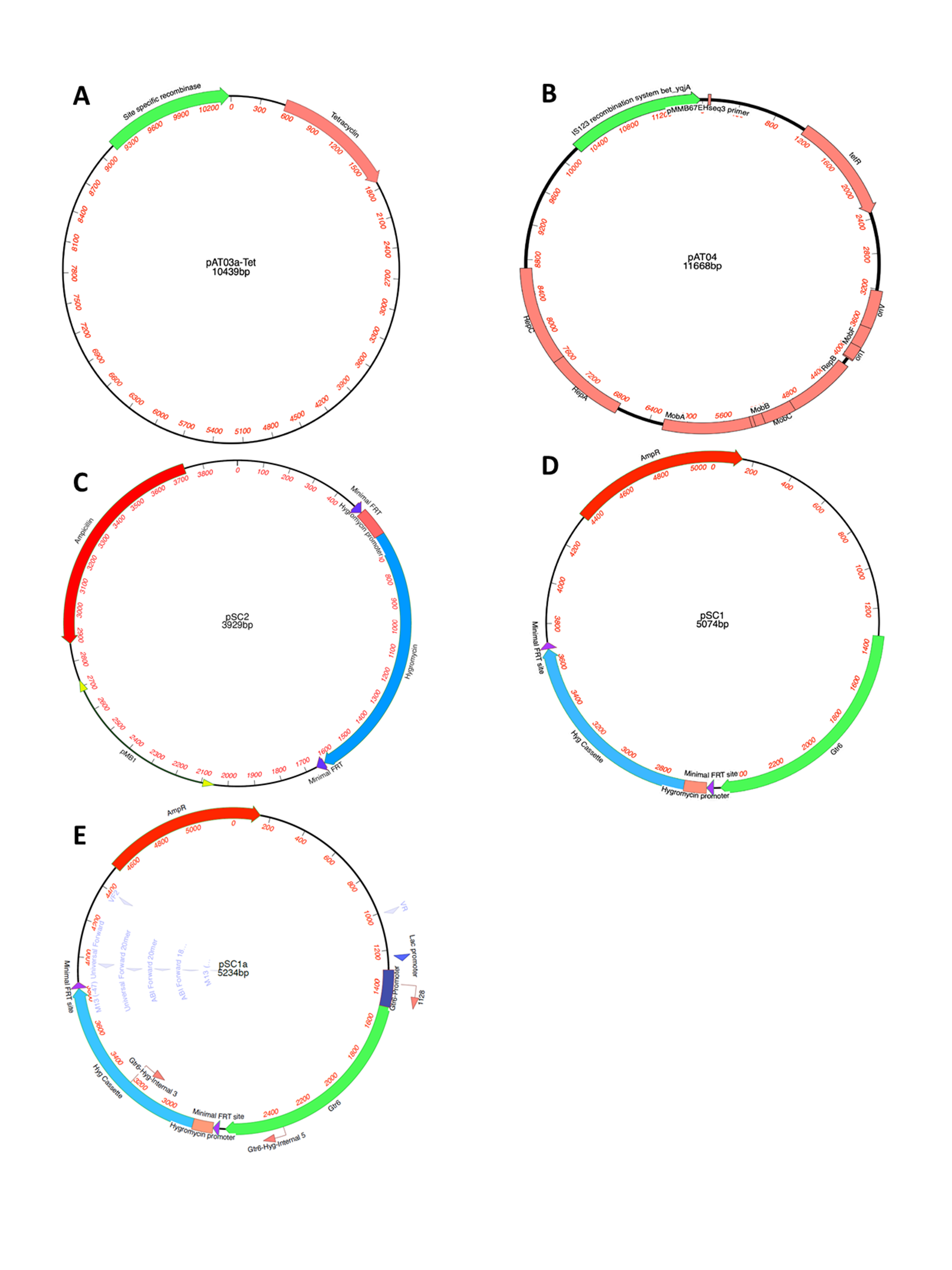

Supplement: S2 Fig — For the generation of the HUMC1::gtr6 mutant, plasmids (A) pAT03a-Tet, (B) pAT04, (C) pSC2, (D) pSC1 and (E) pSC1a were all synthesized as described in the Materials and Methods section. (TIF) [file ppat.1009291.s002.tif]
