# Supplementary material for: Capsule carbohydrate structure determines virulence in Acinetobacter baumannii
Source: PLoS Pathog. 2021 Feb 2;17(2):e1009291. doi: 10.1371/journal.ppat.1009291 (PMC7880449; doi:10.1371/journal.ppat.1009291)
Supplement: S1 Text — Plasmid name, drug marker, function, and origin are listed. (DOCX) [file ppat.1009291.s003.docx]

| **Plasmid** | **Drug marker** | **Use** | **Reference** |
| --- | --- | --- | --- |
| pSIM5 | Chloramphenicol | λ Red recombinase | Datta et al., 2006[1] |
| pBS-kan^r^ | Kanamycin | Kanamycin resistance gene cassette | Lab plasmid |
| pBS-Tet^r^ | Tetracycline | Tetracycline resistance cassette | Lab plasmid |
| pAT03 | Ampicillin | pMMB67EH with FLP recombinase | Tucker et al., 2014[2] |
| pAT03a | Tetracycline | pMMB67EH with FLP recombinase | This study |
| pAT04 | Tetracycline | pMMB67EH with RecAb system | Tucker et al., 2014[2] |
| pSC1 | Hygromycin | corrected gtr6-hygromycin cassette in pUC19 background | This study |
| pSC2 | Hygromycin | Hygromycin cassette with FRT sites | This study |
| pSC1a | Hygromycin | corrected gtr6-hygromycin cassette in pUC19 background for knockout mutant rescue | This study |

**S1 Text. List of all plasmids used in mutant generation.** Plasmid name, drug marker, function, and origin are listed.

**REFRENCES**

1. Datta S, Costantino N, Court DL. A set of recombineering plasmids for gram-negative bacteria. Gene. 2006;379:109-15.

2. Tucker AT, Nowicki EM, Boll JM, Knauf GA, Burdis NC, Trent MS, et al. Defining gene-phenotype relationships in Acinetobacter baumannii through one-step chromosomal gene inactivation. MBio. 2014;5(4):e01313-14.
