# Supplementary material for: Capsule carbohydrate structure determines virulence in Acinetobacter baumannii
Source: PLoS Pathog. 2021 Feb 2;17(2):e1009291. doi: 10.1371/journal.ppat.1009291 (PMC7880449; doi:10.1371/journal.ppat.1009291)
Supplement: S2 Text — The cassette includes the FRT site (red), promoter site for hygromycin (green), and the hygromycin resistance gene (blue). (DOCX) [file ppat.1009291.s004.docx]

**S2 Text. Sequence of hygromycin resistance cassette for mutant generation.** The cassette includes the FRT site (red), promoter site for hygromycin (green), and the hygromycin resistance gene (blue).

GAAGTTCCTATTCTCTAGAAAGTATAGGAACTTCAAAGCCACGTTGTGTCTCAAAATCTCTGATGTTACATTGCACAAGATAAAAATATATCATCATGAACAATAAAACTGTCTGCTTACATAAACAGTAATACAAGGGGTGTTATGACACAAGAGTCACTGTTATTGTTGGATCGCATCGACAGCGACGATTCATATGCCTCTTTGCGTAATGACCAAGAGTTTTGGGAGCCGTTAGCCCGTCGCGCCTTAGAGGAACTGGGTTTGCCTGTACCTCCCGTACTGCGCGTGCCTGGAGAATCGACCAACCCAGTACTTGTGGGGGAGCCCGACCCTGTAATCAAATTATTCGGTGAACATTGGTGTGGCCCTGAATCACTTGCATCAGAATCAGAAGCATATGCGGTCTTGGCTGATGCTCCGGTTCCAGTCCCACGCTTACTGGGGCGTGGAGAGCTTCGTCCCGGTACAGGCGCATGGCCGTGGCCTTACTTAGTTATGAGTCGTATGACTGGGACAACTTGGCGTTCGGCAATGGACGGCACTACTGACCGTAATGCACTGTTGGCACTTGCACGCGAATTGGGACGTGTACTGGGCCGTCTTCACCGCGTGCCACTTACGGGAAACACCGTGTTAACACCTCATTCCGAGGTGTTCCCCGAGCTGCTGCGTGAACGTCGTGCTGCGACAGTTGAAGACCACCGCGGCTGGGGTTACCTGTCGCCTCGCTTATTGGATCGCTTGGAAGACTGGTTGCCAGATGTGGACACGTTGCTGGCGGGGCGTGAACCGCGTTTCGTCCATGGCGATTTGCATGGTACTAATATTTTCGTCGATCTTGCCGCCACTGAGGTCACAGGGATCGTCGATTTCACTGATGTATATGCAGGCGATTCTCGTTATTCGTTAGTACAGCTTCATCTGAACGCGTTTCGTGGAGATCGTGAGATTCTTGCAGCTCTTCTGGATGGGGCACAATGGAAGCGTACCGAAGATTTCGCGCGCGAGCTTTTGGCATTCACTTTCCTGCACGATTTCGAAGTGTTCGAAGAGACGCCCCTTGACTTATCCGGATTTACCGACCCCGAGGAACTGGCGCAGTTTCTTTGGGGACCACCTGACACCGCGCCGGGAGCATGAGAAGTTCCTATTCTCTAGAAAGTATAGGAACTTC
