# Supplementary material for: Capsule carbohydrate structure determines virulence in Acinetobacter baumannii
Source: PLoS Pathog. 2021 Feb 2;17(2):e1009291. doi: 10.1371/journal.ppat.1009291 (PMC7880449; doi:10.1371/journal.ppat.1009291)
Supplement: S3 Text — Primer name, description, and sequence are listed. Underline–first/last 126bp of the gtr6 ORF at the 5’ end. (DOCX) [file ppat.1009291.s005.docx]

| **Primer** | **Use** | **Sequence** |
| --- | --- | --- |
| TetF | 5’ end primer for amplification of the tetracycline resistance gene cassette | CCGGAGGGTGGCGGGCAGGACGCCCGCCATAAACTGCCAGGCATCAAATTAAGCAGAAGGCCATCCTGACGGATGGCCTTTTTGCGTTTCTACAAACTCTAAAGCCACGTTGTGTCTCAAAATCTCT |
| TetR | 3’ end primer for amplification of the tetracycline resistance gene cassette | ATGCATGGCTTTCTACGGGTTCGCTGCGAGTCTTGCCACGCCGAGCACCTGGTCGCTTTCAGAAATCAATCTAAAGTATATATGAGTAAACTTGGTCTGACAGTCAGGTCGAGGTGGCCCGGCTCCAT |
| CHK5Tet | 5’ flanking of antibiotic cassette pAT04, pAT03 and pAT03a plasmids | TGGCCTTTTTGCGTTTCTAC |
| CHK3Tet | 3’ flanking of antibiotic cassette pAT04, pAT03 and pAT03a plasmids | GCTATGTGCAACGGGAATTT |
| pUC19-Gib-F | 5’ Gibson primer for pUC19 amplification | TCCCCGGGTACCGAGCTC |
| pUC19-Gib-R | 3’ Gibson primer for pUC19 amplification | CAGGCATGCAAGCTTGGC |
| Gtr6-Gib-F | 5’Gibson primer for gtr6 amplification | ACGCCAAGCTTGCATGCCTGATGAAAATTGGATTGACAAAATTTAATTATC |
| Gtr6-Gib-R | 3’Gibson primer for gtr6 amplification | ACCCTCCGGCTTAAATACCAATAGTATAAACACTCTTAATTTTATC |
| Hyg-Gib-F | 5’Gibson primer for hygromycin-FRT amplification | TGGTATTTAAGCCGGAGGGTTGCGTTTG |
| Hyg-Gib-R | 3’Gibson primer for hygromycin-FRT amplification | TCGAGCTCGGTACCCGGGGAGTCGCAGCACAGCTCGCT |
| Gtr6-Hyg-5 | 5’ primer for amplification of chimeric gtr6-hygromycin-FRT cassette | ATGAAAATTGGATTGACAAAATTTAATTATCCGGAAATTAGATGTGTTACAACTTCGCAGGAAAATGACTATATAAATTTAAAAAAATATAATATTTATTATTATTTTAATAATATACCA |
| Gtr6-Hyg-3 | 3’ primer for amplification of chimeric gtr6-hygromycin-FRT cassette | AAGCATCTTTCATATAAGCAGCATGTAAAATTTTCATATAAAGACCGGAAAAGTTAAACTTATATATTATAACTAAATATTAGCACCTACGACATCATTATTAGGTACATTAATAACAAGATAACATAGTCGCAGCACAGCTCGCTGGTCCAGAACTGAT |
| Gtr6-Hyg Internal 5 | Primer internal of WT gtr6 gene | ﻿GCTTGTGGTTGCCCTGTAAT |
| Gtr6-Hyg Flanking 3 | 3’ flanking of hygromycin-FRT | AAATCTAAACCATGCCAACCA |
| 1128 | 5’ flanking of gtr6 | TAGATGAATGGTTGGATTGAGTT |
| 1129 | 3’ flanking of gtr6 | CTGAGGAAGCATCTTTCATATAAG |
| Gtr6::kanF | Amplification of kan cassette | ATGAAAATTGGATTGACAAAATTTAATTATCCGGAAATTAGATGTGTTACAACTTCGCAGGAAAATGACTATATAAATTTAAAAAAATATAATATTTATTATTATTTTAATAATATACCAGTCATT*AAAGCCACGTTGTGTCTCAAAATC* |
| Gtr6::kanR | Amplification of kan cassette | TTAAATACCAATAGTATAAACACTCTTAATTTTATCATTATAATAATCAATAGAATGTTCTTTTTTAATACGATTCAATGATTCAACACCTTTATTAATAACAACATTAGGATTTTCATATGCACT*CATTATTCCCTCCAGGTATTAGAA* |
| MutF Outside | For insertion verification of kan cassette | TAGATGAATGGTTGGATTGAGTT |
| MutR Outside | For insertion verification of kan cassette | ATTGCCCGACATTATCGC |
| MutF Kan | For insertion verification of kan cassette | TGCCTCGGTGAGTTTTCTCC |
| MutR Kan | For insertion verification of kan cassette | CTGAGGAAGCATCTTTCATATAAG |
| pSC1-Gib F | Anneals tp pSC1 | ATGAAAATTGGATTGACAAAATTTAATTATCCGGAAATTAGATG |
| pSC1-Gib R | Anneals to pSC1 | AGCTGTTTCCTGTGTGAAATTG |
| Gtr6prom-Gib F | *Gtr6* promoter | ATTTCACACAGGAAACAGCTTAAGGAGATTAAGTAATAGTAAAGTTATAATAATATTAG |
| Gtr6prom-Gib R | *Gtr6* promoter | TTTGTCAATCCAATTTTCATATAGTAACCTTAAAAAATAGTTAAATCAC |

**S3 Text. List of all primers used for mutant generation.** Primer name, description, and sequence are listed. Underline – first/last 126bp of the *gtr6* ORF at the 5’ end.
